# Spherulitic Lead Calcium Apatite Minerals in Lead Water Pipes Exposed to Phosphate-Dosed Tap Water

**DOI:** 10.1021/acs.est.2c04538

**Published:** 2023-03-15

**Authors:** Jeremy D. Hopwood, Helen Casey, Martin Cussons, Porsha Knott, Paul N. Humphreys, Hayley Andrews, Jenny Banks, Stephen Coleman, John Haley

**Affiliations:** † School of Applied Sciences, University of Huddersfield, Huddersfield HD1 3DH, U.K.; ‡ Faculty of Science and Engineering, Manchester Metropolitan University, Manchester M15 6BH, U.K.; § Yorkshire Water, Yorkshire Water Services, Western House, Halifax Road, Bradford BD6 2SZ, U.K.

**Keywords:** lead pipe, plumbosolvency, spherulite, apatite, tap water, phosphate, mineral, crystallization

## Abstract

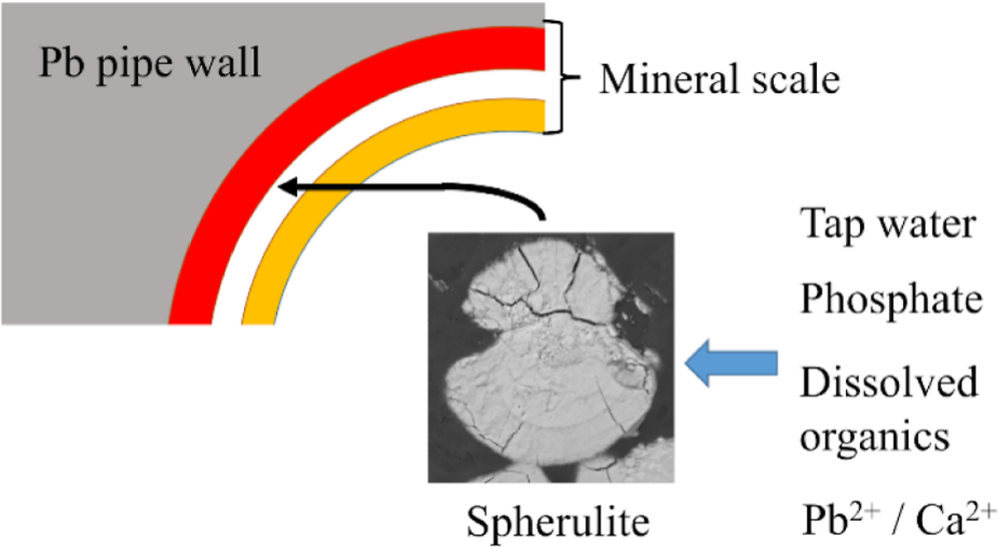

Phosphate dosing is the principle strategy used in the United Kingdom to reduce the concentration of lead in tap waters supplied by lead water pipes. The mechanisms of phosphate-mediated lead control are not fully understood, but solid solutions of lead calcium apatite are thought to play an important role. This study investigated the microstructure of a lead pipe, supplied with high-alkalinity tap water, in which the lead calcium apatite crystals were spherulitic having rounded and dumb-bell-shaped morphologies. XRD, Fourier transform infrared spectroscopy, optical microscopy, Raman spectroscopy, scanning electron microscopy, and energy-dispersive spectroscopy showed that the lead pipe had a well-established inner layer of litharge; a middle layer containing lead calcium apatite spherulites, plumbonacrite, and some hydrocerussite; and an outer layer containing iron, lead, phosphorus, calcium, silicon, and aluminum. It was found that spherulitic lead calcium apatite could be grown in the laboratory by adding hydrocerussite to synthetic soft and hard water-containing phosphate, chloride, and citrate ions at pH 5.5 but not when the citrate was absent. This suggests that dissolved organic molecules might play a role in spherulite formation on lead water pipes. These molecules might inhibit the formation of lead calcium apatite, reducing the effectiveness of phosphate dosing in lead water pipes.

## Abstract 2

This study investigates the spherical shaped lead phosphate crystals that are sometimes found in lead water pipes when phosphate is added to tap water to control lead release and goes on to discuss their wider application within the context of the mineral scale.

## Introduction

In the United Kingdom, many homes are supplied with tap water that has passed through a lead service pipe, which connects the home to the mains pipe in the street. Precise figures are not available, but housing data^1,2^ indicates that approximately one-third or 9.5 million of all U.K. properties are connected in this way.

The harmful effects of lead are well known, and in the United Kingdom, the current regulatory limit for a random daytime sample of 1 L of tap water, taken from a customer’s kitchen tap, is 10 μg/L total lead.^3^ The principle strategy adopted by all U.K. water utilities to control lead at customers’ taps is to dose the tap water with phosphate (orthophosphate).^4−6^ Most U.K. utilities use 3–6 mg/L phosphate (1–2 mg/L as P) at a pH between 7 and 8. The practice of phosphate dosing began in the 1970s and 80s with a gradual increase in the proportion of supplies treated until 2000 when dosing became widely adopted. Since 2000, compliance with the 10 μg/L limit increased from 95.0 to 99.5% (Figure S1).^7^ Therefore, from a regulatory and public health perspective, the practice of phosphate dosing has been very successful.^8^

The original theory was that phosphate dosing would promote the formation of highly insoluble lead phosphate minerals pyromorphite [Pb_5_(PO_4_)_3_Cl] and hydroxylpyromorphite [Pb_5_(PO_4_)_3_OH],^9^ which would lower the concentration of lead. It was thought that a layer of these minerals would grow on top of the existing lead carbonate layer, possibly acting as a physical barrier against further release of lead and would take up to a year to provide full protection.^10^

Most of the studies on the mineral scales in lead water pipes have been undertaken in the United States and Canada, where a greater variety of methods are used for mitigating corrosion. Ongoing studies of lead water pipes from Midwestern and Northeast states of the United States have found that approximately 25% of operators use orthophosphate, another 25% use blended phosphate (a mixture of orthophosphate and polyphosphates), and the remainder use pH and alkalinity control.^11^ The most common minerals observed in 300 pipe samples were litharge (PbO), plattnerite (β-PbO_2_), cerussite (PbCO_3_), and hydrocerussite [Pb_3_(CO_3_)_2_(OH)_2_].^11−14^ Lead phosphate minerals occurred in only some of the cases in which orthophosphate and blended phosphate have been added to tap waters.^14^ In this case, hydroxylpyromorphite [Pb_5_(PO_4_)_3_OH], lead phosphate [Pb_9_(PO_4_)_6_], and a mixed lead–calcium phosphate mineral [Pb_*x*_Ca_5–*x*_(PO_4_)_3_(Cl,OH)] were observed.^12^ A similar set of minerals have been observed in pipes from the United Kingdom except that plattnerite has not been observed.^15^

**Figure 1:**
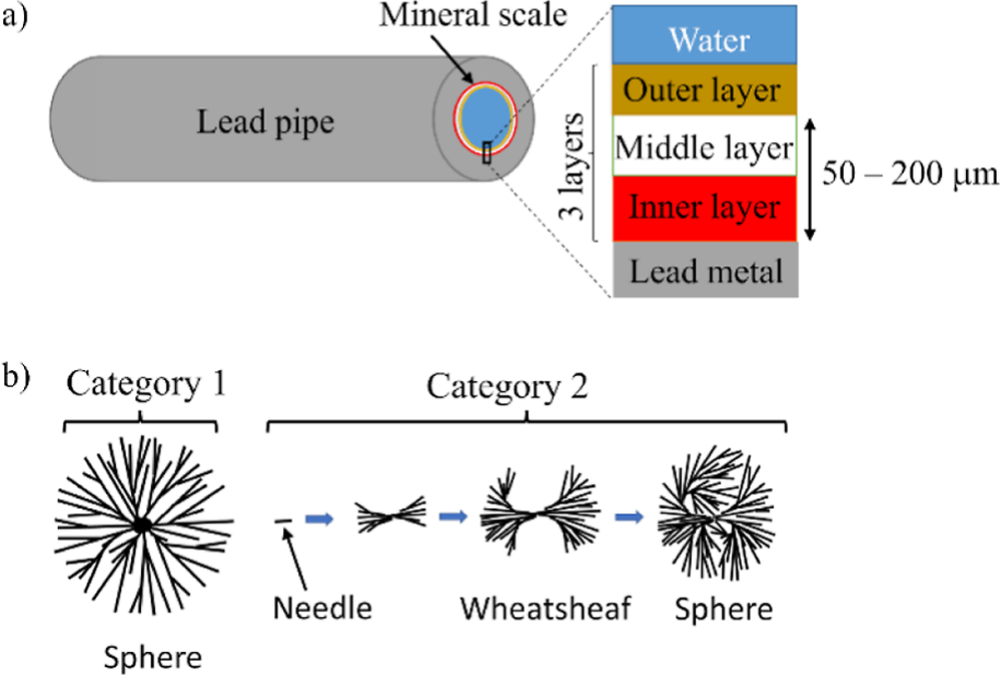
Schematic diagrams of the lead pipe scale and spherulites. (a) Model of the three-layer structure of mineral scales in U.K. lead water pipes, as envisaged by the authors. (b) Structure of spherulites in cross-section as proposed by Gránásy et al.^21^ who defines two types of spherulites: category 1 and category 2. The dark lines represent the needles or fibers.

The chemistry of lead pipe mineral scale is complex, and the mechanisms of corrosion and mineralization are not well understood. Some lead pipes are over 100 years old, and their scale might reflect conditions that are very different from those of today. In the United Kingdom, the formation of lead phosphate minerals must have occurred within the last 20–40 years, the time period of phosphate dosing. Recent studies^11,16,17^ have shown that the mineral phases within the scale are spatially distributed within discrete layers. For the purpose of this study, a simplified schematic diagram has been created by the authors (Figure 1a). The inner layer, which is adjacent to the lead metal, usually comprises litharge (PbO), which is recognized by its orange/red color. The middle layer, adjacent to the inner layer, is where the lead carbonates and lead phosphate minerals are located. The outer layer is a loosely adhered layer, which can display different surface textures, patterns, and colors. It is often rich in Fe, Al, O, Si, P, Ca, and Pb and may be non-crystalline (amorphous).^12^ Plattnerite, which is orange/red but darker than litharge, can occur in any one of the three layers (in North American pipes).^11,12,18^

Observing lead minerals within the mineral scale is often difficult because of their small size. Medium resolution scanning electron microscopes, which are commonly used, cannot resolve the morphologies of nano-sized particles. However, recent analyses by the authors have revealed unusual spherical or rounded shaped crystals in some of the U.K. lead water pipes that are readily seen by scanning electron microscopy (SEM). Preliminary analyses of these micron-sized crystals indicated that they were spherulitic in morphology and comprised lead calcium apatite. Crystals that look spherulitic have been reported in a small number of lead pipe rig studies,^19^ although they were not identified as spherulites. Spherulites are not uncommon in crystal growth but are not widely reported in the literature. They are polycrystals with a radial structure of needles or fibers that branch at small angles^20^ and can be loosely grouped into category 1 spherulites, where branching occurs from a central core, and category 2 spherulites, where branching occurs off a central needle (Figure 1b).^21^ Although the name implies that they are spherical, the term includes spheres, dumb-bell-shaped structures, and wheatsheaf-shaped structures.

The occurrence of lead calcium apatite crystals with unusual shapes implies that the mineral might not be growing in an expected manner. The thermodynamic and kinetic properties of these spherulites might differ from those of single crystals with well-defined planar crystal faces. This difference is of interest to U.K. water utilities who would like to have a better understanding of how phosphate mitigates lead in tap water.

This study focused on a pipe obtained from the city of Kingston upon Hull, East Yorkshire, that had been supplied with high-alkalinity water and contained spherulitic crystals. A comprehensive analysis of both the mineral scale and the spherulites within was undertaken using powder X-ray diffraction (XRD), Fourier transform infrared (FTIR) spectroscopy, optical microscopy (OM), SEM, energy-dispersive spectroscopy (EDS), and Raman spectroscopy. A small number of lead water pipes from other locations that contained spherulites were also investigated. Spherulitic crystals of lead calcium apatite were grown in the laboratory from the dissolution and reprecipitation of the lead carbonate mineral hydrocerussite in synthetic soft and hard tap waters that contained a mixture of calcium, phosphate, chloride, and citrate ions.

The aims of this study were to understand the phenomena of spherulite formation on lead water pipes to discuss this in the wider context of the lead pipe mineral scale and to understand more about how phosphate influences the chemistry of the lead pipe mineral scale. The objectives were to (1) characterize the structure of the mineral scale; (2) confirm whether the mineral scale comprised an inner, a middle, and an outer layer; (3) characterize the structure of the spherulites; (4) confirm whether the unusual rounded crystals in lead water pipes were spherulitic and comprised lead calcium apatite; (5) determine whether spherulitic lead calcium apatite could be grown in the laboratory; and (6) determine factors that give rise to lead calcium apatite spherulites.

## Materials and Methods

### Extraction of Lead Pipe and Sample Preparation

The lead pipe used in this study was a communication pipe that connected the mains pipe under the street to the stop tap in the pavement and was owned by the utility. The supply pipe, which connected the stop tap in the pavement to the home was not analyzed.

The lead communication pipe was extracted in 2010 from a terraced property (built in 1901–1911) in Kingston upon Hull, East Yorkshire (postcode HU3), and analyzed between 2015 and 2020. It was drained on site and sealed at both ends with plastic bags before being sent to the laboratory, whereupon it was air-dried. It was then stored dry at room temperature and 40–60% humidity. A rachet pipe cutter with a clean blade was used to cut the pipe into shorter transverse sections (length 1.5 cm). Smaller longitudinal sections were then cut with a clean, stainless steel, utility knife (Stanley).

The transverse sections were embedded in under vacuum using the Struers Epofix cold mounting resin. The purpose of the vacuum was to remove air bubbles in order to improve contact between the pipe and the resin. The embedded samples were covered and left to harden in air for a day at room temperature. Cross-sections of the pipe were then ground and polished. The sequence was (1) manual polishing with 240 SiC paper on a granite grinding plate with a gentle flow of tap water and rotating by 90° every 30 s, (2) manual polishing with p600 SiC paper for 5 min on a polishing wheel, (3) manual polishing with p1200 SiC paper for 5 min, (4) gently rubbing the surface with a piece of selvyt cloth coated in 1 μm diamond suspension spray, and (5) rinsing the surface with tap water and then methanol before being air-dried. The tap water used during this sequence was not from the sampling site but from the mains supply at the University of Huddersfield. The water contained phosphate, which would have limited the amount of mineral dissolution (1 mg/L as P, Alk 10 mg/L CaCO_3_, pH 7.2).

Samples of the mineral corrosion scale for XRD and FTIR analyses were removed by firmly scraping the insides of the dried pipes with a clean stainless steel spatula until some of the underlying lead metal was visible. Studies in the United States advocate collecting the scale at successive depths in order to differentiate the minerals found in the different layers. This technique of successive scrapping has been used on U.K. lead water pipes (unpublished work) and has shown that the size of the litharge peak increases with depth. However, other changes in mineral composition have not been seen. Therefore, complete scrapings rather than successive scrapings were used on the pipes in this study. It is worth noting that the thickness of the mineral scale on U.K. lead water pipes extends over a short distance (typically 20–200 μm for the inner and middle layer), making it hard to remove and separate discrete layers.

### Water Quality

The water in the city of Kingston upon Hull is a high-alkalinity tap water extracted from a chalk bedrock. The pipe from Hull had been exposed to this type of water for over 100 years. During this time, chlorine was always used as a disinfectant, and flocculants were never used. Phosphate dosing was initiated in 2001. Since then, the average alkalinity and average pH of the water have been 240 mg/L CaCO_3_ and pH 7.1, respectively. A more detailed analysis is given in Table S1.

### Analytical Techniques

Powder XRD was carried out using a Bruker D2 PHASER fitted with a LYNXEYE detector and Cu Kα radiation (=1.5418 Å). Samples of scraped mineral scale were ground in isopropanol using a pestle and mortar and loaded into the center of a silicon crystal sample holder with no cavity. Scans were performed between the diffraction angles 10 and 60° 2θ. FTIR of the scraped mineral scale was undertaken using a Thermo Scientific Nicolet iS5 spectrometer. Scans were performed between 500 and 2000 cm^–1^.

Microscopy was undertaken looking down on the mineral scale in a plan view and across the mineral scale in a cross-sectional view. Optical microscopy was carried out using a digital Keyence VHX-2000 microscope equipped with a VHX-J250 lens. SEM and elemental analysis (EDS) of the lead pipe minerals scale were performed using a FEI Quanta 250 FEG microscope equipped with an Oxford Instruments X-Max detector, a Zeiss Supra 40VP FEG microscope equipped with a EDAX Inc, an Apollo 40 SDD, and a Zeiss Evo MA10 tungsten filament microscope equipped with an Oxford Instruments X-Max detector. The images from the different microscopes were taken at an accelerating voltage of 10–20 kV at a working distance of 4.5–12 mm. Plan view images were taken in the secondary electron mode, and cross-sectional images were taken in the backscattered mode. EDS point analyses and area analyses were recorded to see what elements were present. EDS maps were recorded to see where elements were concentrated. The lighter shades were probably background radiation (further explanation is given in Text S1). The samples of pipe (transverse and longitudinal) were attached to aluminum stubs with a conducting carbon sticky tape, and the sides were coated with the conducting paint. Where necessary, the samples were then coated with a thin layer of gold/palladium (15–20 nm). Raman microscopy was performed on the polished sections of the pipe using a Thermo Scientific DXR Raman microscope. A green laser of wavelength 532 nm was used. Cross-sections of the mineral scale were very sensitive to the Raman laser, and there was a playoff between power, resolution, and damage. Each point was photobleached for 2 min after which five 30 s frames were taken. A relatively low power of 0.4 mW was found to give optimum results, although the signal-to-noise ratio for many of the peaks was high.

### Citric Acid Speciation Calculations

The speciation of lead and citrate ions in equilibrium with the lead carbonate mineral hydrocerussite was modeled at 25 °C using the ion speciation software PHREEQC Version 3.3.^22^ The database used was minteq.dat version 4.0,^23^ which was modified slightly to include more recent values from the 2009 IUPAC technical report on Pb^2+^ ions.^24^ The input files are shown in Table S2 and Figure S2.

### Laboratory Synthesis of Spherulites

The reagents used were of analytical grade and are listed in Table S3. The lead carbonate mineral hydrocerussite was prepared using the method of Giammar^25^ by simultaneous addition of lead nitrate solution (50 mL, 0.015 M) and sodium hydrogen carbonate solution (50 mL, 0.010 M) to ultrapure water (500 mL, 18.2 MΩ·cm) with rapid stirring. The two solutions were added dropwise over 30 min, and the pH was continually adjusted to pH 8.0 by the addition of 0.1 M carbon dioxide-free NaOH. The resulting white suspension was filtered off using a 0.45 μm Millipore filter, and the damp solid was transferred to a sample vial containing 50 mL of ultrapure water. The mass of hydrocerussite in the vial was 200 mg (based on >95% yield). Powder XRD of a sample of a dried solid gave a single large peak at 2θ = 34.0° corresponding to the {110} planes of hydrocerussite (Figure S16a). The single large peak was due to the orientation of the hydrocerussite crystals. These were shown by SEM to be thin hexagonal plates of diameter 1–3 μm, stacked with their large faces on top of each other (Figure S16b).

The spherulites were prepared by adding 25 mL of the hydrocerussite suspension (100 mg of solid) into a 1 L solution of synthetic soft water or hard water and at pH 5.5. The soft and hard waters were made by dissolving CaCO_3_ powder (calcite) in ultrapure water while bubbling with CO_2_ gas. To these were added stock solutions of NaH_2_PO_4_, NaCl, and trisodium citrate (Na_3_C_6_H_5_O_7_), and the pH was increased to 5.5 by bubbling with air to remove CO_2_(g). The soft water contained 20 mg/L dissolved CaCO_3_, and the hard water contained 200 mg/L CaCO_3_. Both finished solutions contained 30 mg/L phosphate (10 mg/L as P), 10 mg/L chloride, and 0.5 mM citrate (equivalent to 36 mg/L organic carbon). The suspensions of hydrocerussite in soft and hard water were sealed in 1 L of high-density polyethylene sample bottles with minimum air space. They were then stirred with a magnetic stirrer and sampled after 1 day. The samples were dried and analyzed by XRD, FTIR, SEM, and EDS. The relatively low pH of 5.5, the high concentration of phosphate of 30 mg/L (10 mg/L as P), and the high concentration of citrate (0.5 mM) were chosen to maximize the likelihood of precipitating spherulites in a small period of time, not to mimic the conditions within a lead water pipe. Control experiments were also undertaken in which the citrate was omitted.

## Results

This section considers the analyses of the lead pipe mineral scale, the lead pipe spherulites, and the laboratory-grown spherulites.

### Mineral Scale

#### Visual Observations and Optical Microscopy of the Mineral Scale

A light brown, partly fragmented mineral scale with cracks and valleys was observed within the lead pipe (Figure S3). Some of the fragmentation must have been artifactual due to the drying down process and to the stresses on the pipe during cutting, and some may have occurred when the pipe was in operation.

#### XRD and FTIR of the Mineral Scale

XRD of complete scrapings of the mineral scale indicated that litharge (α-PbO), lead–calcium apatite [Pb_*x*_Ca_5–*x*_(PO_4_)_3_(OH,Cl)], hydroxyapatite [Ca_5_(PO_4_)_3_OH], hydrocerussite [Pb_3_(CO_3_)_2_(OH)_2_], and/or plumbonacrite [Pb_5_(CO_3_)_3_O(OH)_2_] were present. The minerals were identified by comparing the pattern (Figure S4a) with the standard patterns (Figures S4b and S5). The most prominent peaks were from the {101} and {110} crystal planes of litharge. Less prominent were the peaks at 2θ = 22.1 and 30.8°, which were similar but not the same as those associated with phosphohedyphane [Ca_2_Pb_3_(PO_4_)_3_Cl], which has peaks at 2θ = 21.9 and 30.9° (Figure S4b). Both hydrocerussite and plumbonacrite appeared to be present, but it was difficult to separate the two sets of peaks. Peaks for plattnerite (β-PbO_2_), which have been observed in pipes in North America supplied with chlorinated tap water,^11,13^ were not observed.

The presence of carbonate and phosphate components in the bulk mineral scale was confirmed by FTIR (Figure S6). The doublet at 541 and 586 cm^–1^ was characteristic of lead apatite minerals.^15^

#### OM and SEM Images of the Mineral Scale in Plan View

Figure S7a,b shows the same area of mineral scale in plan view by OM and SEM. The area contains a valley, which may have formed from a piece of scale flaking off. The regions of inner, middle, and outer layers are marked as I, M, and O, respectively. In the OM image (Figure S7a), the red inner layer can be seen at the base of the valley, the white middle layer can be seen at the edges of the valley, and the brown outer layer can be seen on the surface. The border of the valley is marked in Figure S7b by the white line.

Magnified images of the boxed areas in Figure S7a,b are shown in Figure S7c–e. The outer layer (Figure S7c) contained irregular-shaped aggregates of diameter <5 μm, whose fine structure was not resolved by SEM. The middle layer (Figure S7d,f) contained spherulitic-shaped particles that were 5 μm in diameter. These had a textured surface. The inner layer contained interlocking particles of size 0.2–5 μm (Figure S7e). They were plate like, and some had a hexagonal outline suggestive of crystals. It was not clear whether these particles were on the surface of the inner layer or whether they were part of the inner layer.

#### OM, SEM, EDS, and Raman Spectroscopies of the Mineral Scale in Cross-Section

**Figure 2:**
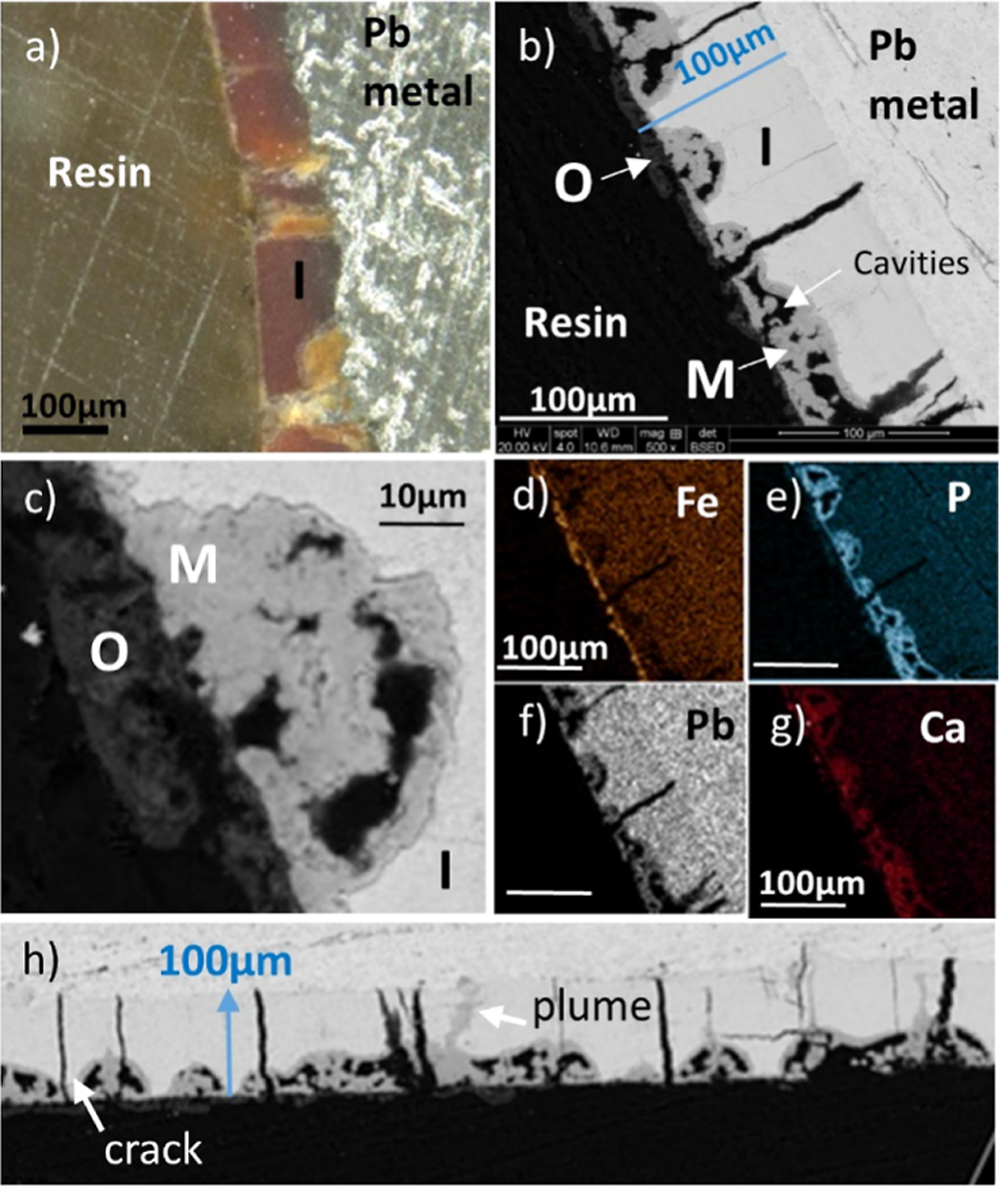
Microscopic images showing the structure of the mineral scale in cross-section. (a) OM image showing a well-pronounced orange/red inner layer. (b,c,h) Backscattered SEM images and EDS maps from a different area. For EDS (d–g), the concentration ∝ brightness in each map only. The images b–g all correspond to areas within image h. The inner, middle, and outer layers are labeled I, M, and O, respectively.

The inner, middle, and outer layers I, M, and O, respectively, were clearly visible when the scale was viewed in cross-section by OM and SEM (Figure 2), but unlike the structure shown in the schematic diagram in Figure 1, the inner and middle layers were not parallel sided. The inner layer, which was orange/red when viewed by OM and light gray in the SEM, was the most prominent of the three layers, with a thickness of up to 100 μm. The side of the inner layer next to the lead metal was planar, but the other side was pitted. These pits, which were 30–40 μm deep, contained the middle layer interspersed with large cavities. The outer layer was the thinnest layer with a depth of 15 μm. The scale also contained plume-shaped regions from the base of the pits to the lead metal and cracks that penetrated the entire mineral scale. The cracks may have grown along the plumes. A more precise schematic diagram of the three-layer structure of the mineral scale is given in Figure S8.

The elemental maps (Figure 2d–g) showed Fe to be concentrated in the outer layer and P and Ca to be concentrated in the middle layer. More information about the maps and how they were interpreted are given in Text S1. Raman spectroscopy provided further evidence of the distribution of the minerals within the three layers. The spectra coupled with the OM images showed that phosphate and carbonate were concentrated in the middle layers and that litharge was concentrated in the inner layer (Figures S10 and S11).

Individual EDS spectra across the mineral scale (Figure S9) indicated that Al and Si were also present in the outer layer; that Sn was a minor component of the metal, inner layer, and middle layer; and that there was no P in the inner layer.

### Lead Pipe Spherulites

**Figure 3:**
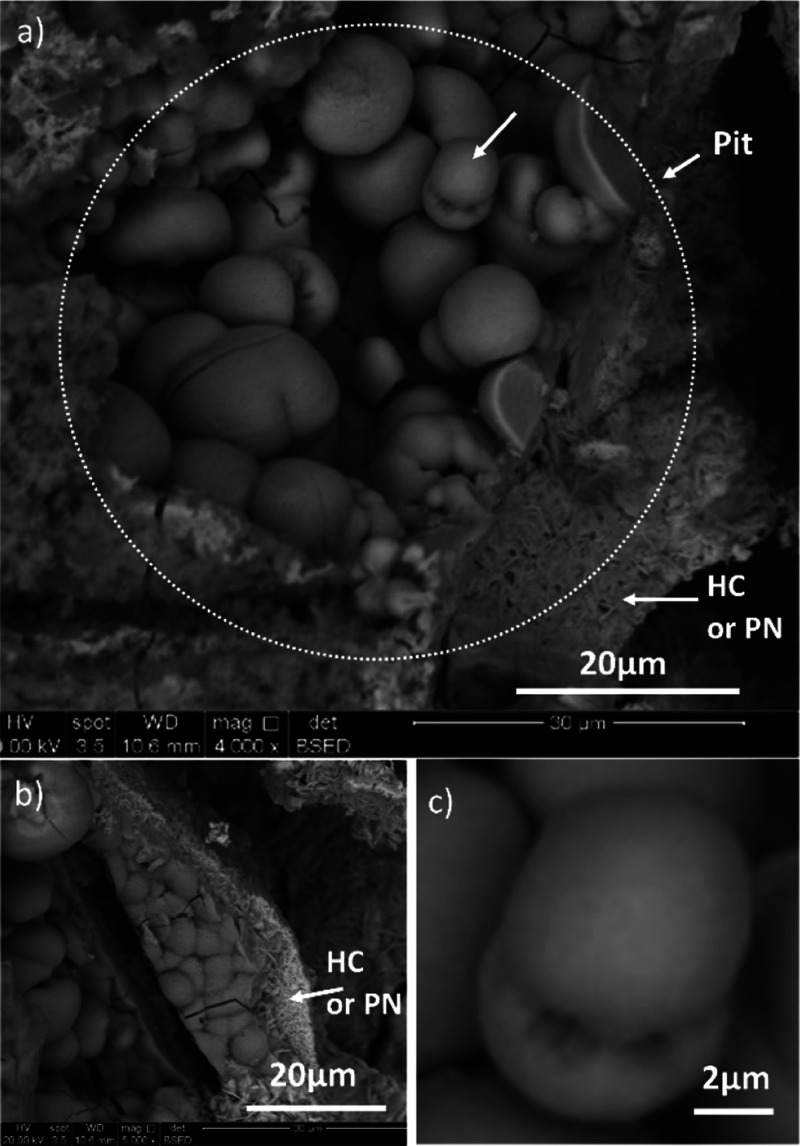
Secondary electron SEM images of spherulitic crystals in the middle layer of the lead pipe mineral scale. The nearby plate-like crystals in “a” and “b” were probably hydrocerussite HC or plumbonacrite PN, as peaks for both were observed in XRD (Figure S4). Figure “c” is an enlargement of the dumbbell-shaped spherulite in figure “a” (arrowed).

**Figure 4:**
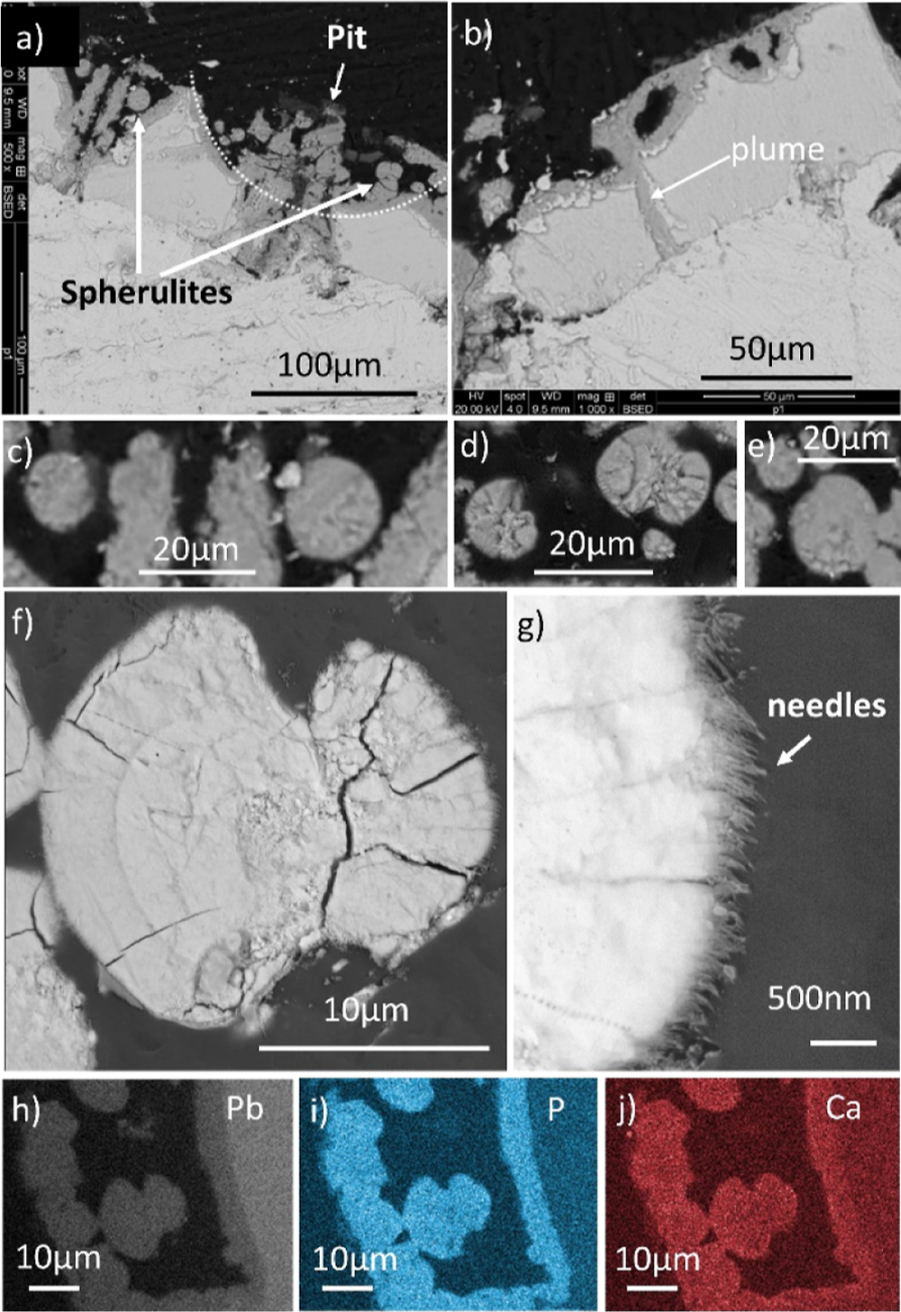
Backscattered SEM images and EDS elemental maps of the spherulites and the surrounding mineral scale in cross-section. (a,b) Two regions with pits, (c) enlargement of the spherulites in “a”, (d–f) more examples of spherulites, (g) enlargement of the right side of the spherulite in “f”, and (h–j) EDS maps of the dumbbell spherulite in “f” together with the surrounding scale (concentration ∝ brightness in each map only). See also Figure S13.

Close inspection of the middle layer by SEM in plan view (Figure 3) revealed numerous rounded particulates. Spheres and the figures of eight, dumbbell, morphologies of diameter 5–20 μm were present. These were also observed by SEM when the middle layer was viewed in cross-section (Figures 4 and S13). Although the surfaces of the particulates looked smooth in plan view, they were found to comprise many small needle-like protrusions in cross-section (Figure 4g). The shapes of the rounded crystals together with the needle-like protrusions indicated that they were spherulitic.

The elemental maps and spectra of a cross-section through a dumbbell-shaped spherulite (Figures 4 and S13b) indicated that it contained Pb, P, Ca, and O, which is consistent with the spherulite being made of lead calcium apatite. Al was also detected. The elements were not spatially segregated, indicating that the composition of the spherulite was uniform throughout. Plate-like crystals were often in close proximity to the spherulites, which were probably the crystals of hydrocerussite and/or plumbonacrite (labeled HC/PN in Figure 3), as detected by XRD.

### Laboratory Grown Spherulites

**Figure 5:**
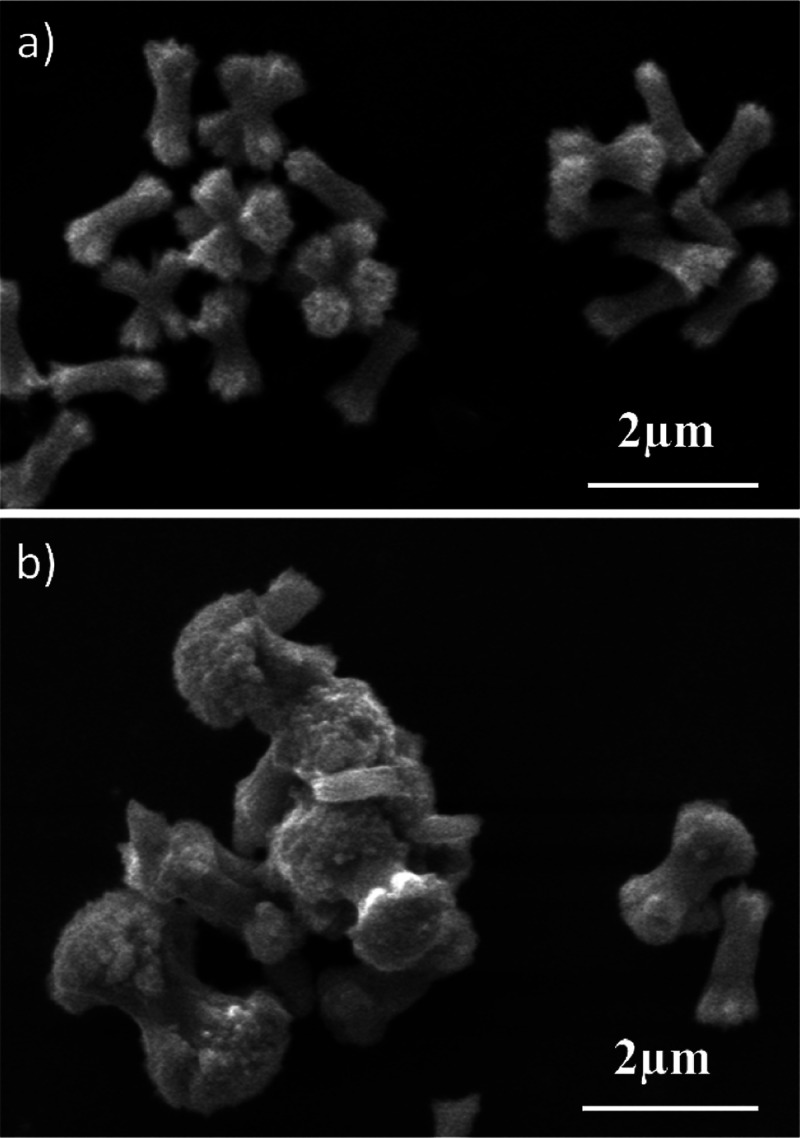
Secondary electron SEM images of spherulites grown in the laboratory in (a) soft water and (b) hard water. The crystals were grown from the dissolution of hydrocerussite at pH 5.5, phosphate = 30 mg/L (10 mg/L as P), chloride = 10 mg/L, citrate = 0.5 mM (36 mg/L as organic C), and sampled after 1 day.

Wheatsheaf-shaped crystals were observed in experiments conducted using soft and hard waters containing citric acid (Figure 5). Those from the soft waters were 1.5 μm long with small diverging ends and those from the hard waters were 2–3 μm long with more pronounced diverging ends. Fibers were sometimes seen emanating from the ends of the crystals. The crystals were therefore spherulitic in structure. The spherulites were smaller to those seen in the lead water pipe. Elemental EDS spectra for the spherulites contained peaks for Pb, P, and Ca (Figure S14).

The XRD patterns for spherulites grown in synthetic soft and hard waters were similar to each other (Figure S15) and closely resembled those of pyromorphite and hydroxypyromorphite standards (Figures S4b and S5). It was not possible to tell which was the best fit without more detailed analysis. Unlike the standards, the peaks were broad, indicating that small nanosized particles or misaligned domains were present, which is consistent with the needle-like structure found in spherulites. Hydrocerussite crystals, which were added at the beginning of both experiments (Figure S16), were absent and had completely dissolved.

The control experiments in which citrate was omitted from the soft and hard waters did not produce spherulites. Instead, very small crystals were observed that appeared to grow off the remaining hydrocerussite crystals (Figures S17b and S18b). These crystals were not spherulitic. The XRD patterns indicated that the small crystals comprised lead apatite (Figures S17a and S18a).

## Discussion

### Spherulites

Crystals are often described as having morphologies defined by planar crystal faces. For example, sodium chloride readily forms cubic crystals. At high saturations, dendrites can form, such as those associated with snowflakes. The sodium chloride cubes and the dendritic snowflakes are single crystals. However, some crystals are polycrystalline, which means they comprise many single crystals attached to each other, and spherulites belong to this group. Spherulite crystals have been found in polymers, alloys, biological materials, organic compounds, liquid crystals, and minerals.^21^

It is generally accepted that the spherulitic shape (Figure 1c) is a result of small-angle branching of needles or fibers.^20,21,26−29^ These secondary branches are slightly misaligned from the primary needle or fiber and over time multiple sub-branches grow. All the needles and fibers are uniaxial, i.e., they are elongated along the same crystal axis. However, because they are misaligned, they are sometimes called non-crystallographic branches or small-angle branches. In some spherulites, the elongated crystallites radiate from a central core to give a spherical shape (category 1). However, in others, there is no core, and instead, the misalignment of the adjacent elongated crystallites and branches causes the fibers to spread out like a fan, giving rise to wheatsheaf-shaped crystals, dumbbells, and spheres (category 2).^21^ The two types are shown in Figure 1b.

A growth front nucleation mechanism in which slightly misaligned nuclei grow into branches has been used to explain the shapes of the spherulites.^21^ Computational models of the solid–liquid interface using phase field theory have been used to investigate this mechanism and applied to spherulitic growth in metal solidification and polymer crystallization.^30,31^ However, non-classical crystal growth mechanisms have also been proposed in which the needles and fibers are regarded as mesocrystals formed by the aggregation and alignment of nano-sized particles.^32−36^ The two competing theories have led to heated debate.^37^

Spherulites are often found in systems in which there is a high thermodynamic driving force for crystallization coupled with a kinetic barrier. The latter gives rise to slower than expected crystal growth. Examples of kinetic barriers include the presence of impurities, surface inhomogeneities, and lattice defects.

Examples of mineral spherulites include calcium carbonate (vaterite) spherulites that grow at moderate supersaturation without impurities,^35^ strontium sulfate (celestite) spherulites that grow on calcium sulfate (gypsum) crystals as they dissolve,^38^ fluorapatite spherulites that grow in the presence of additives such as citric acid or gelatin,^39,40^ hydroxyapatite spherulites that grow in a collagen gel,^41^ and calcium carbonate (calcite) spherulites that grow in the feces of ruminant herbivores^42^ and fish.^43^ Spherulitic minerals have also been grown in solutions containing bacteria,^44^ where control of crystal growth has been attributed to the release of an extracellular material such as polysaccharides (EPSs). Spherulites can form in any crystal system, but the likelihood is increased if the crystal is insoluble and if it grows with a needle-like morphology.^20^

### Lead Pipe and Laboratory-Grown Spherulites of Lead Calcium Apatite

The spherulites observed in this study were not an isolated case, and various spherulite morphologies of lead calcium apatite spherulite have been observed by the authors in other lead water pipes from the United Kingdom. The SEM and EDS images of some of these spherulites are shown in Figures S19–S21, and others are reported elsewhere.^17^

Lead calcium apatites have the potential to form spherulites because they are very insoluble in the presence of phosphate and because they tend to form needles. However, for spherulites to form, the needles must undergo small-angle branching. In other systems, this has been attributed to the adsorption of inorganic or organic impurities^27,45^ or to surface inhomogeneities. The latter might arise from the lattice strain caused by the incorporation of Ca^2+^ and Pb^2+^ ions of different size or by the incorporation of other ions such as CO_3_^2–^ and Al^3+^.

The laboratory experiments were undertaken to acquire a better understanding of spherulite crystal growth. The aim was not to mimic conditions on the lead pipe but to explore whether lead calcium apatite spherulites could be grown. Hydrocerussite was used as the source of Pb because lead carbonate minerals occur in the mineral scale and because they are more soluble than lead calcium apatite minerals, when phosphate is present. In addition, hydrocerussite provided a surface for reprecipitation reactions. The concentration of phosphate (PO_4_ = 30 mg/L) was 5–10 times that found in the lead pipe (PO_4_ = 3–6 mg/L) because the laboratory time available for the experiments was short. For the same reason, the pH was reduced to pH 5.5 to enhance the rate of dissolution of the hydrocerussite crystals. Calcium was added as dissolved calcium carbonate, which meant that both Pb^2+^ and Ca^2+^ ions were present. Citric acid was used because it had been shown to promote spherulitic growth in calcium carbonate^27^ and fluorapatite precipitation.^39^ The laboratory experiments indicated that spherulites of lead calcium apatite could be grown when hydrocerussite was added to soft and hard waters containing citric acid at pH 5.5. The control experiments without citric acid did not produce spherulites, and so citric acid was a factor in spherulite crystal growth. It is not clear at this stage whether a mixture of soluble Ca and Pb is needed for spherulite formation at pH 5.5, but it might be important at the higher pH values found in tap waters.

One consequence of using citric acid at 0.5 mM was that a large amount of lead would have been solubilized. Citric acid is a small triprotic organic acid, H_3_Cit, with three carboxylic groups and one alcohol group (Figure S22) that forms stable complexes with divalent metal ions. At pH 5.5, and in the presence of lead, most of the citrate forms PbCit^–^ ions. Solubility and speciation calculations using PHREEQC (Figure S18) indicate that the amount of lead solubilized in a suspension of hydrocerussite in soft water at pH 5.5 will be ≈100 mg/L and that in hard water will be ≈60 mg/L. Such high concentrations of soluble lead are unlikely in the mineral scale of a lead water pipe. This is one reason why reducing the citric acid concentration and increasing the pH will be needed in future experiments to reduce the concentration of soluble lead. Spherulites might still grow under these conditions, especially if strong adsorption of the citrate molecules onto the crystal surface is more important than the concentration of PbCit^–^ ions.

The results from the citric acid experiments suggest that dissolved organic molecules are important for spherulite formation in lead water pipes. Possible candidates include humic and fulvic acids that are present in tap waters. These molecules, which contain carboxylic groups, are already known to affect the concentration of lead in tap water.^46^ Other candidates include organic molecules such as extracellular polymeric substances, EPS, that are released by bacteria, as biofilms have been observed on lead water pipes.^47^ Spherulitic lead phosphate crystals have been grown in the laboratory in the presence of aerobic *Acetobacter* sp.,^48^ although the media was rich in organics, which is not the case for tap waters. Regardless of the source, dissolved organic molecules can act as crystal growth inhibitors. In lead pipes, they might reduce the rate of lead calcium apatite growth, thereby reducing the effectiveness of phosphate dosing. This is why the spherulites are so interesting as they might help to identify dissolved organic molecules that effect the chemistry of lead calcium apatite and hence effect the release of lead. Without further study, it is not yet possible to say when the spherulites grew on the lead pipe. They may have grown soon after the lead pipe was first exposed to phosphate in 2001 when the dissolution equilibria for the existing lead carbonate phases would have changed.

Recently, the importance of lead pipe sample methodology for ensuring accurate microscopy results has been raised.^49^ With this in mind, there is a small possibility that the spherulites grew when the pipe was transported after it was drained on site. However, a simple calculation of the pipe geometry and the number of moles of lead and phosphate (Calculation S1) indicates that there was insufficient concentration of soluble phosphate in a 1 mm film of tap water (a worst-case scenario) to account for the amount of lead calcium apatite seen. Furthermore, spherulites have since been seen in pipes that are emptied and immediately air-dried.

### Heterogeneous Nature of the Lead Pipe Mineral Scale

If the mineral scale was homogeneous, then only one mineral phase would be present, and according to thermodynamics, this would be the most insoluble mineral phase. In lead pipes supplied with phosphate-dosed tap waters, lead calcium apatite minerals would grow, eventually replacing the more soluble minerals until only lead calcium apatite was present. However, lead water pipes such as those in the United Kingdom are heterogeneous with a three-layer structure, and the amount of lead calcium apatite in the scales of these pipes varies considerably from pipe to pipe. Full conversion to a lead phosphate mineral has not taken place despite 20 years of phosphate dosing.

The inner layer on this lead pipe was very prominent comprising the highly soluble mineral litharge, PbO. The mechanism for PbO growth is not known. It might be solution-mediated or involve a solid-state transition from lead metal to lead oxide. The absence of PO_4_^3–^ and Ca^2+^ ions in the inner layer is consistent with the latter. The fact that one of the two prominent XRD peaks was broad and the other narrow, which suggested that the crystals of PbO were anisotropic in shape, either thin plates or thin needles.

The middle layer was a mixture of lead carbonate minerals (hydrocerussite and plumbonacrite) and lead calcium apatite spherulites. The lead carbonate minerals are much more soluble than the lead calcium apatites when phosphate is present, which means that the carbonates should dissolve and lead calcium apatite should precipitate. This is a precipitation dissolution process. The addition of phosphate causes the water in contact with the lead carbonate minerals to become supersaturated with respect to lead calcium apatites and these minerals grow. This removes soluble lead, causing the lead carbonate minerals to become undersaturated and dissolve.

The development of the layered structure can be attributed to interface coupled dissolution precipitation processes (ICPD) in which nucleation and growth of the more insoluble mineral occurs on or near a dissolving crystal. Precipitation and dissolution are spatially coupled, and one mineral replaces by another. This process, also called a mineral replacement reaction, has been extensively studied in geochemistry.^50−52^ Studies on ICPD tend to use relatively large crystals, typically 0.1–5.0 mm in diameter. Because of this, the more insoluble mineral may have time to form a protective outer layer on the surface of the dissolving mineral before complete dissolution takes place, thereby reducing the rate of dissolution. This outer layer can slow the diffusion of ions to and from the reaction interface, especially if its porosity is low. In the case of lead water pipes, the combined inner and middle layers are typically 50–200 μm thick, and so protective surface layers can also develop. This would explain why the litharge, PbO, inner layer is stable despite being highly soluble. It follows that the porosity of the mineral scales may be important. The difference in porosity may explain why some U.K. lead pipes contain more lead calcium apatite minerals than other pipes even when they are exposed to similar pH, alkalinity, and phosphate concentrations. More porous layers would result in more conversion of the soluble lead oxide and lead carbonate minerals to lead calcium apatite.

Other heterogeneous features of the mineral scale were the presence of mineralized channels (plumes) through the inner layer, which connected the middle layer to the lead metal, and the presence of cracks and cavities. All are features of minerals undergoing ICPD. In ICPD, the channels and cracks provide an easier route by which ions can diffuse through the mineral layers. The simplest explanation for the cavities is that the rate of dissolution was faster than the rate of precipitation. However, it is not clear when this event took place. The cavities may predate phosphate dosing. Cavities have also been observed in lead pipe scales from North America, where they are associated with the dissolution of PbO_2_. The cavities are interesting as they might provide local environments in which the water chemistry is different from that of the bulk water flowing through the pipe. Cavities have been observed in mineral formations that are in contact with biofilms,^53^ and therefore, it is conceivable that bacteria might play a role in lead water pipes. The presence of phosphate in tap water would help in this regard.

### Environmental Implications of Spherulites

Spherulites represent a pathway by which phosphate is mineralized in the mineral scale of the lead pipe, and this mineralization may account for the reduction in lead concentration seen after phosphate dosing.

This study confirmed that the unusual rounded, wheatsheaf- or, dumb-bell-shaped crystals seen in some lead water pipes, after phosphate dosing, were spherulitic, comprising a radial arrangement of needles. The spherulites were present in the middle layer of the mineral scale and must have formed by mineral replacement reactions in which lead carbonate or lead oxide minerals, formed prior to phosphate dosing, were dissolved and more insoluble lead calcium apatite minerals were precipitated. Dissolution precipitation reactions were replicated in the laboratory and showed that spherulites of lead calcium apatite could be grown by adding the lead carbonate mineral hydrocerussite to soft and hard waters containing phosphate, chloride, and citric acid at pH 5.5. However, no spherulites formed when citric acid was absent, which indicated that dissolved organic molecules might play a role in spherulitic lead calcium apatite formation on lead water pipes.

Future work will investigate whether laboratory spherulites can be grown under conditions closer to those found in tap waters (pH 7–8, 3–6 mg/L phosphate). It will also look at the impact of supersaturation and reaction rate on spherulite formation and will identify the origin of the dissolved organic molecules, whether they are from humic and fulvic acids in tap water or from biofilms attached to the walls of the lead pipe.

## Supporting Information Available

Lead compliance in the United Kingdom; composition of the tap water; stability constants for citrate; PHREEQC input files; list of chemicals; three-layer model of pipe scale; additional XRD, EDS, OM, SEM, Raman, and FTIR data; interpretation of EDS data; speciation diagrams for the Pb–Ca–citrate system; and calculation showing that spherulites were not an artifact of drying (PDF)
