# Supplementary material for: Spherulitic Lead Calcium Apatite Minerals in Lead Water Pipes Exposed to Phosphate-Dosed Tap Water
Source: Environ Sci Technol. 2023 Mar 15;57(12):4796–805. doi: 10.1021/acs.est.2c04538 (PMC10061917; doi:10.1021/acs.est.2c04538)
Supplement: Supplementary file 1 — es2c04538_si_001.pdf [file es2c04538_si_001.pdf]

Supporting Information

Spherulitic lead calcium apatite minerals in lead water pipes exposed to phosphate dosed tap water.

Jeremy Hopwood[1,*], Helen Casey[1], Martin Cussons[1], Porsha Knott[1], Paul Humphreys[1], Hayley Andrews[2], Jenny Banks[3], Stephen Coleman[3], John Haley[3].

[1]School of Applied Sciences, University of Huddersfield. [2]Manchester Metropolitan University

[3]Yorkshire Water, Yorkshire Water Services, Western House, Halifax Road, Bradford.

33 pages 23 figures 3 tables 1 calculation 1 text description

Contents

Figure S1. Change in lead compliance in England and Wales S3
Table S1. Composition of tap water that flowed through the pipe from Hull. S3
Table S2. Stability constants for citrate, calcium citrate and lead citrate. S4
Figure S2. PHREEQC input files used to create speciation graphs in Figure S23. .... S4
Table S3. Information about the chemicals. S5
Figure S3. Low magnification images of the lead pipe mineral scale S6
Figure S4. XRD patterns S7
Figure S5. Standard XRD spectra from JCPDS, ICDD and RRUFF databases. S8
Figure S6. FTIR spectra of scrapings of the lead pipe mineral scale. S9
Figure S7. Microscope images in plan view of a valley in the mineral scale S10
Figure S8. The 3-layer model of the lead pipe mineral scale S11
Figure S9. EDS analysis of the mineral scale S12
Text S1. Interpretation of the EDS Maps S13
Figure S10. Raman spectroscopy of the mineral scale, analysis 1 .... S14
Figure S11. Raman spectroscopy of the mineral scale, analysis 2 .... S15
Figure S12. Raman spectra of standards and pure lead minerals. S16
Figure S13. Additional backscattered SEM images of lead pipe spherulites. S17
Figure S14. Elemental EDS spectra and SEM images of laboratory grown spherulites. S18
Figure S15. XRD spectra of laboratory grown spherulites. S19
Figure S16. XRD pattern and SEM image of hydrocerussite used to make spherulites. S20
Figure S17. XRD pattern and SEM image of crystals from the soft water control expts. S21
Figure S18. XRD pattern and SEM image of crystals from the hard water control expts. S22
Figure S19. OM and SEM images of spherulites observed on an additional pipe S23

Figure S20. SEM images of spherulitic lead calcium apatites observed on 3 more pipes...S24
Figure S21. EDS spectra of spherulites from pipes P1, P3, P6 and P7...............................S25
Figure S22. Structure of the citrate ion. S26
Figure S23. Speciation diagrams for 0.5mM citrate in equilibrium with in soft and hard waters containing hydrocerussite. S27
Calculation S1. Calculation showing that the spherulites are not caused by sample preparation. S28

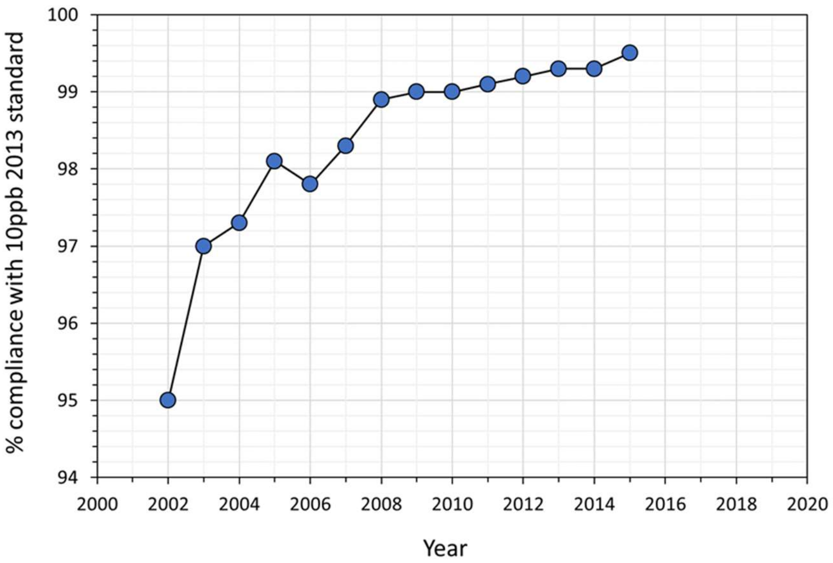

Figure S1. Change in lead compliance in England and Wales with Pb_{TOT} < 10 ppb[1]. In the U.K. compliance is measured by taking random daytime samples from customer properties. Customers are chosen at random and samples are taken between 9am and 5pm on weekdays.

Table S1. Composition of tap water that flowed through the pipe from Hull at the time the pipe was sampled (2010). Only the main species / parameters are shown. The values came from random daytime samples from customer properties. The calculated values were derived from the full composition data together with PHREEQC.

pH	**7.1**
Total Alkalinity, mg/l $CaCO_3$	240
Calc. Alkalinity, mg/l $CaCO_3$	277
Ca, mg/l	130
PO_4^{3-}, mg/l	2.4
Calc. DIC, mg/l	76.8
Calc. Ionic strength, M	0.01
TOC, mg/l	0.5 – 1.0

Table S2. Stability constants for citrate, calcium citrate and lead citrate. The constants are from Minteq 4 [2], which originated from the NIST standard reference database number 46, version 2 [3].

Equilibrium	constant
$H_3Cit \rightleftharpoons H^+ + H_2Cit^-$	$pk_1 = 3.128$
$H_2Cit^- \rightleftharpoons H^+ + HCit^{2-}$	$pk_2 = 4.76$
$HCit^{2-} \rightleftharpoons H^+ + Cit^{3-}$	$pK_3 = 6.396$
$Ca^{2+} + Cit^{3-} \rightleftharpoons CaCit^-$	logk = 4.87
$Ca^{2+} + Cit^{3-} + H^+ \rightleftharpoons CaHCit$	logk = 9.26
$Ca^{2+} + Cit^{3-} + 2H^+ \rightleftharpoons CaH_2Cit^+$	logk = 12.257
$Pb^{2+} + Cit^{3-} \rightleftharpoons PbCit^-$	logk = 7.27
$Pb^{2+} + 2Cit^{3-} \rightleftharpoons PbCit_2^{4-}$	logk = 6.53

```
SOLUTION 1. Soft water
   temp      25
   pH       5.5
   pe       4
   redox     pe
   units     mg/kgw
   density   1
   Alkalinity 0.4 meq/kgw
   Ca        8
   Citrate   0.5 mmol/kgw
   -water    1 # kg
EQUILIBRIUM_PHASES 1
   Hydrocerrusite 0 10

SOLUTION 2. Hard water
   temp      25
   pH       5.5
   pe       4
   redox     pe
   units     mg/kgw
   density   1
   Alkalinity 4.0 meq/kgw
   Ca        80
   Citrate   0.5 mmol/kgw
   -water    1 # kg
EQUILIBRIUM_PHASES 1
   Hydrocerrusite 0 10
```

Figure S2. PHREEQC input files used to create the speciation graphs shown in Figure S23.

Table S3. Information about the chemicals. These were used to make the synthetic tap water, hydrocerussite and lead calcium spherulites in the laboratory.

**Chemical**	**Supplier**	**Purity**	**State**
Sodium hydroxide 50% in aqueous solution Reag. Ph. Eur. 1081406 carbonate free (1L)	VWR		Solution
Lead Nitrate Solution (0.1M)*<br>EP Grade, Reagecon™	FisherSci	99.0%	Solution
Carbon dioxide gas in a pressurised cylinder. UN1013	BOC	>99.9%	Gas
Calcium carbonate	FisherSci	99.3%	Solid
Sodium hydrogen carbonate	FisherSci	≥99%	Solid
Sodium dihydrogen phosphate monohydrate	Acros	99%	Solid
Sodium chloride	FisherSci	>99.9%	solid
Anhydrous trisodium citrate	Acros	98%	Solid

*The lead nitrate was purchased as a solution so that lead nitrate powder was not used in the laboratory. This reduced the health and safety risks associated making lead minerals.

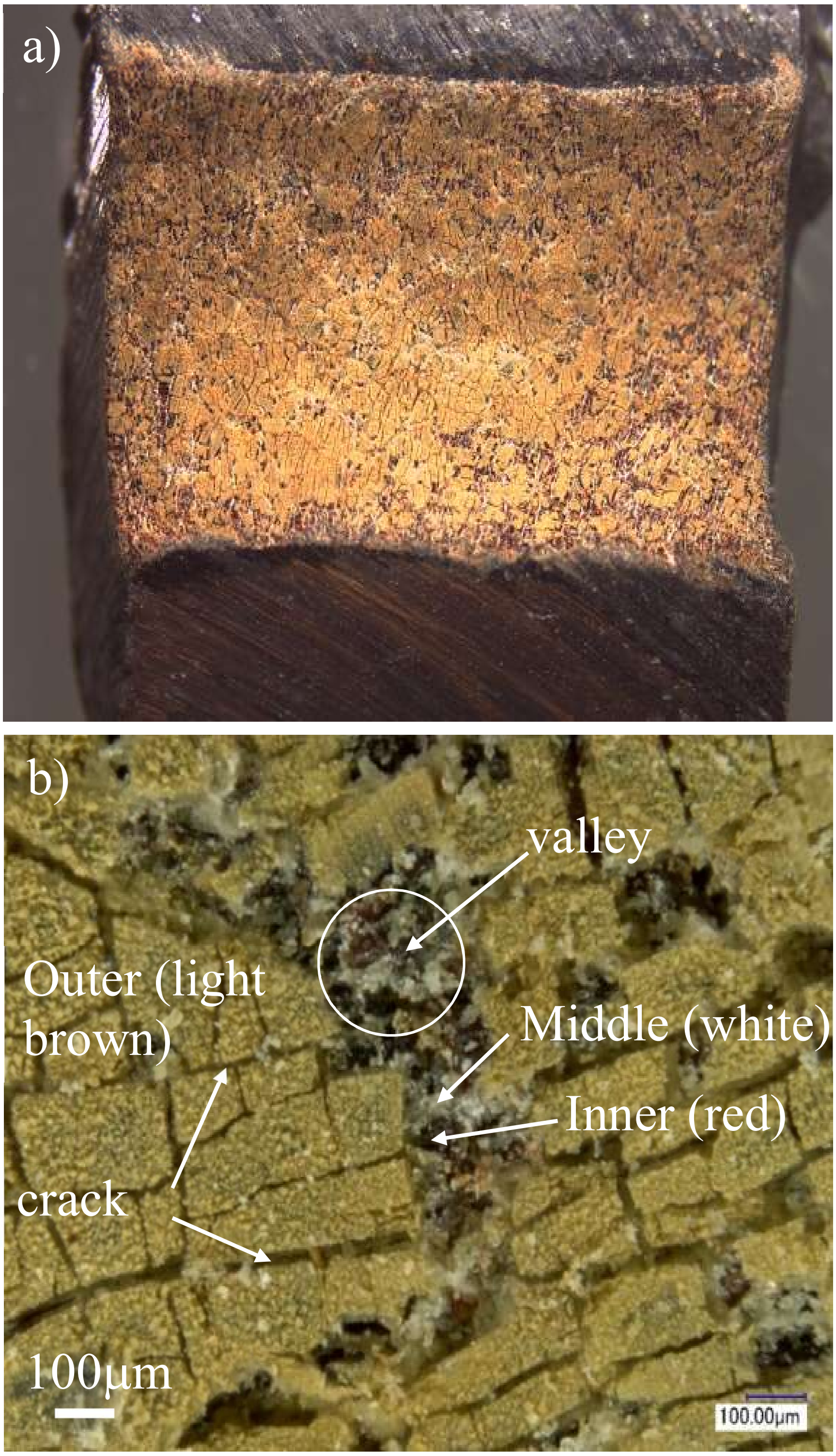

Figure S3. Low magnification images of the lead pipe mineral scale. a) camera image of the inside of a piece of lead pipe. b) reflected light optical microscope image of pipe in 'a' The light brown regions show the outer layer. The white regions are the middle layer and the dark red regions are due to the presence of litharge (the inner layer). The valleys containing the white and dark red regions are where fragments of scale have broken off.

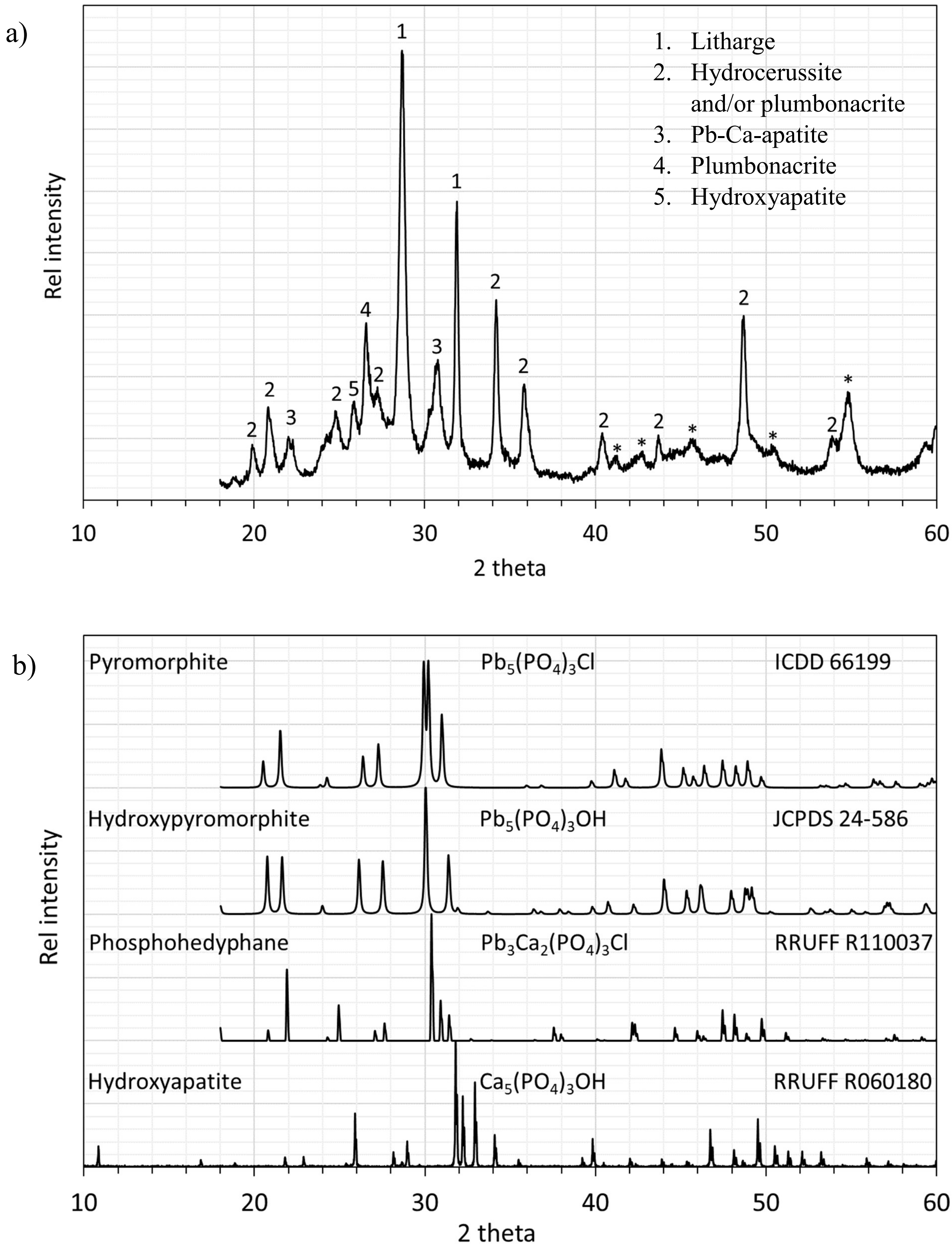

Figure S4. XRD patterns. a) XRD of the lead pipe mineral scale. This is the same pattern as that shown in Figure 4 except that it is for 2θ = 10 – 60. Peaks labelled * were unknown. b) Standard XRD patterns of Pb, Pb/Ca and Ca apatites taken from the ICDD, JCPDS and RRUFF[4] databases.

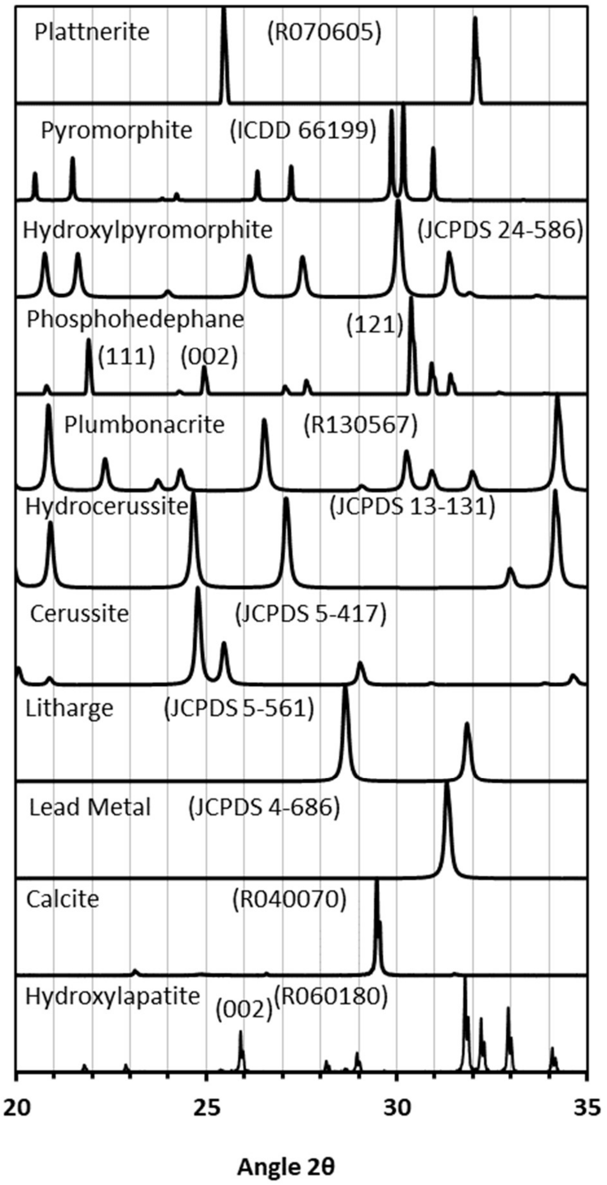

Figure S5. Standard XRD spectra from JCPDS, ICDD and RRUFF[4] databases.

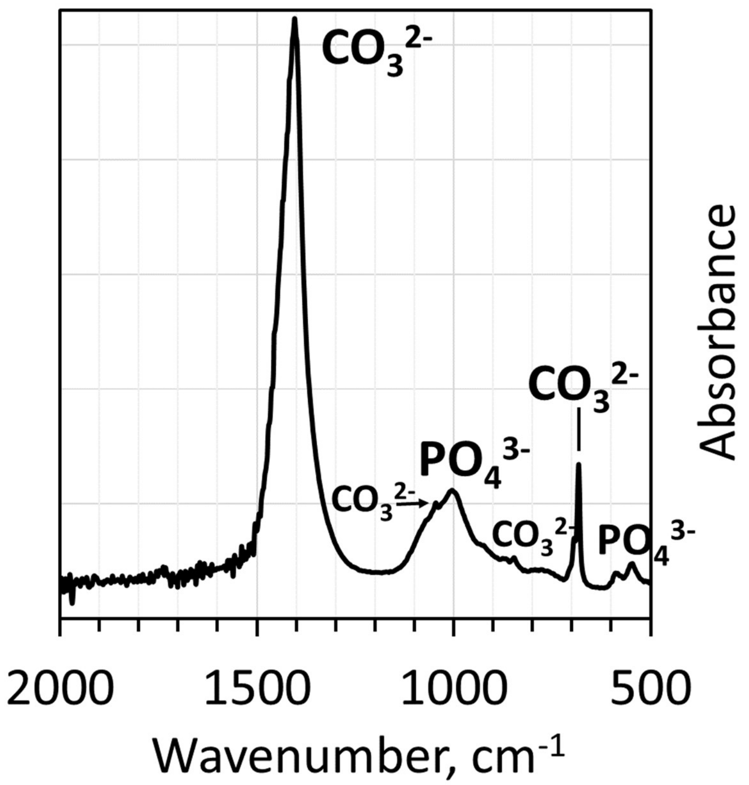

Figure S6. FTIR spectra of scrapings of the lead pipe mineral scale.

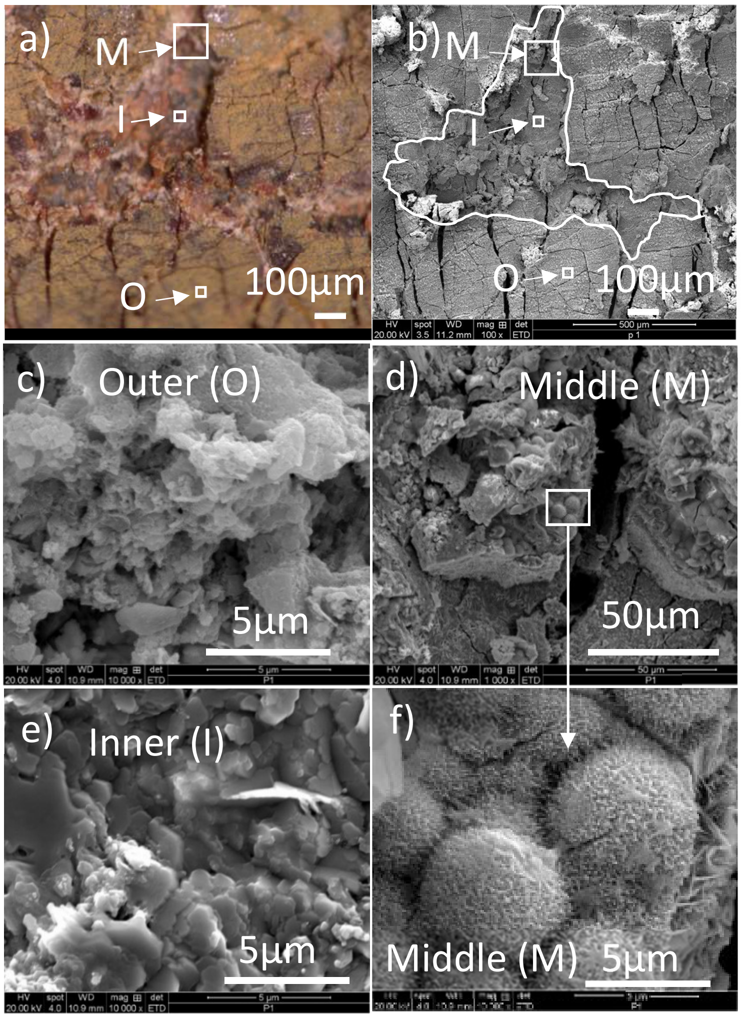

Figure S7. Microscope images in plan view of a valley in the mineral scale showing the I inner, M middle and O outer layers. a,b) low magnification OM and secondary electron SEM images, c-f) enlargements of the boxed regions shown in Fig a and Fig b.

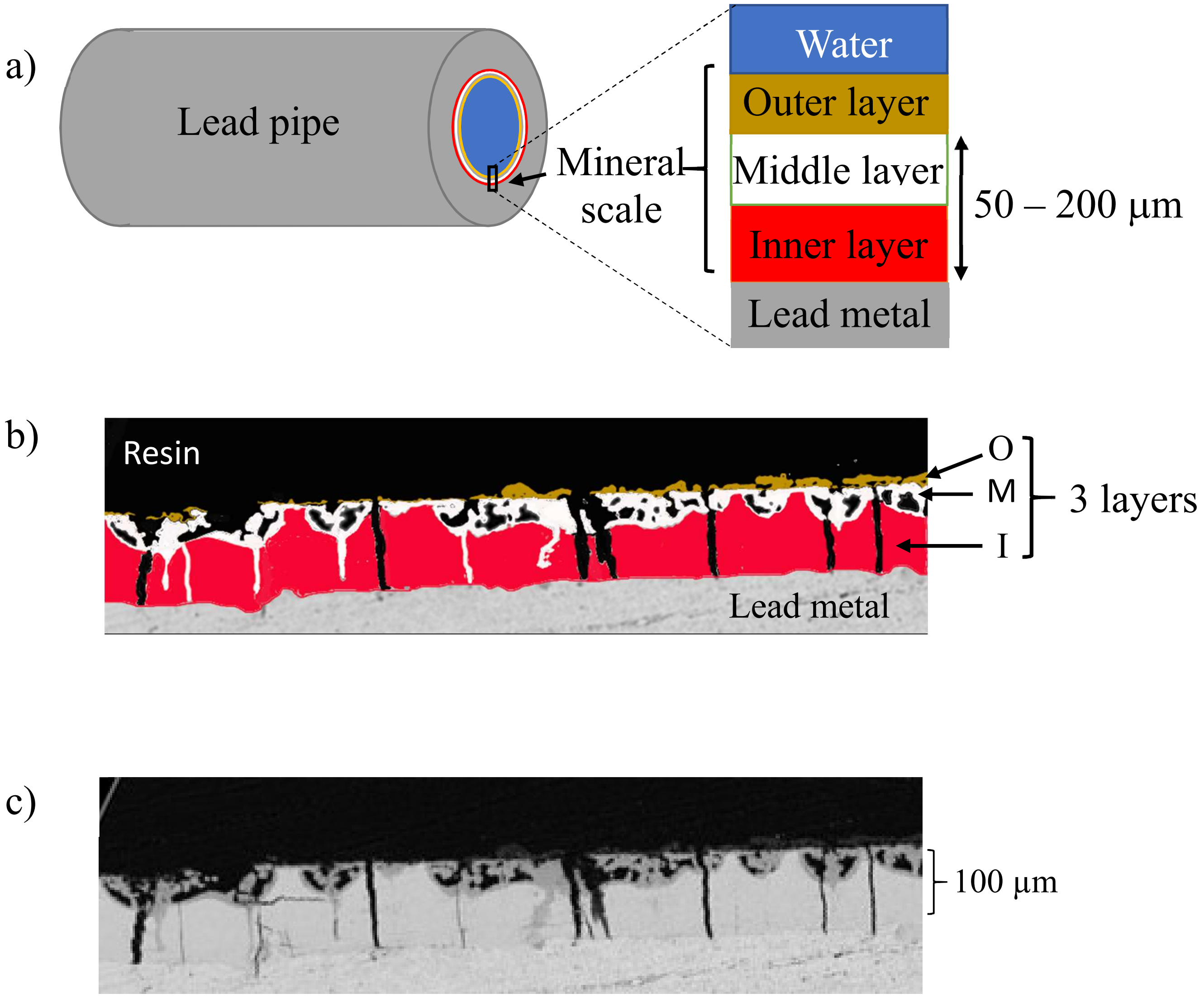

Figure S8. The 3-layer model of the lead pipe mineral scale. a) Schematic diagram of the mineral scale. The outer layer comprises material deposited from the water supply network. It is often rich in iron and light brown in colour. The middle layer of pipes supplied with phosphate dosed water is a mixture of lead carbonate and lead phosphate minerals and is white in colour. The inner layer comprises PbO and has a red colour. b) Actual back scattered SEM image of the polished section of the pipe studied here, with digital colour applied to the layers. The spherulites of lead calcium apatite in the pipe studied here were present within the white middle layer together with lead carbonate minerals. The locations of the different minerals were identified by combining the results from XRD, FTIR, OM, SEM, EDS and Raman spectroscopy. c) The actual back scattered image of the polished section of pipe mineral scale before applying colour.

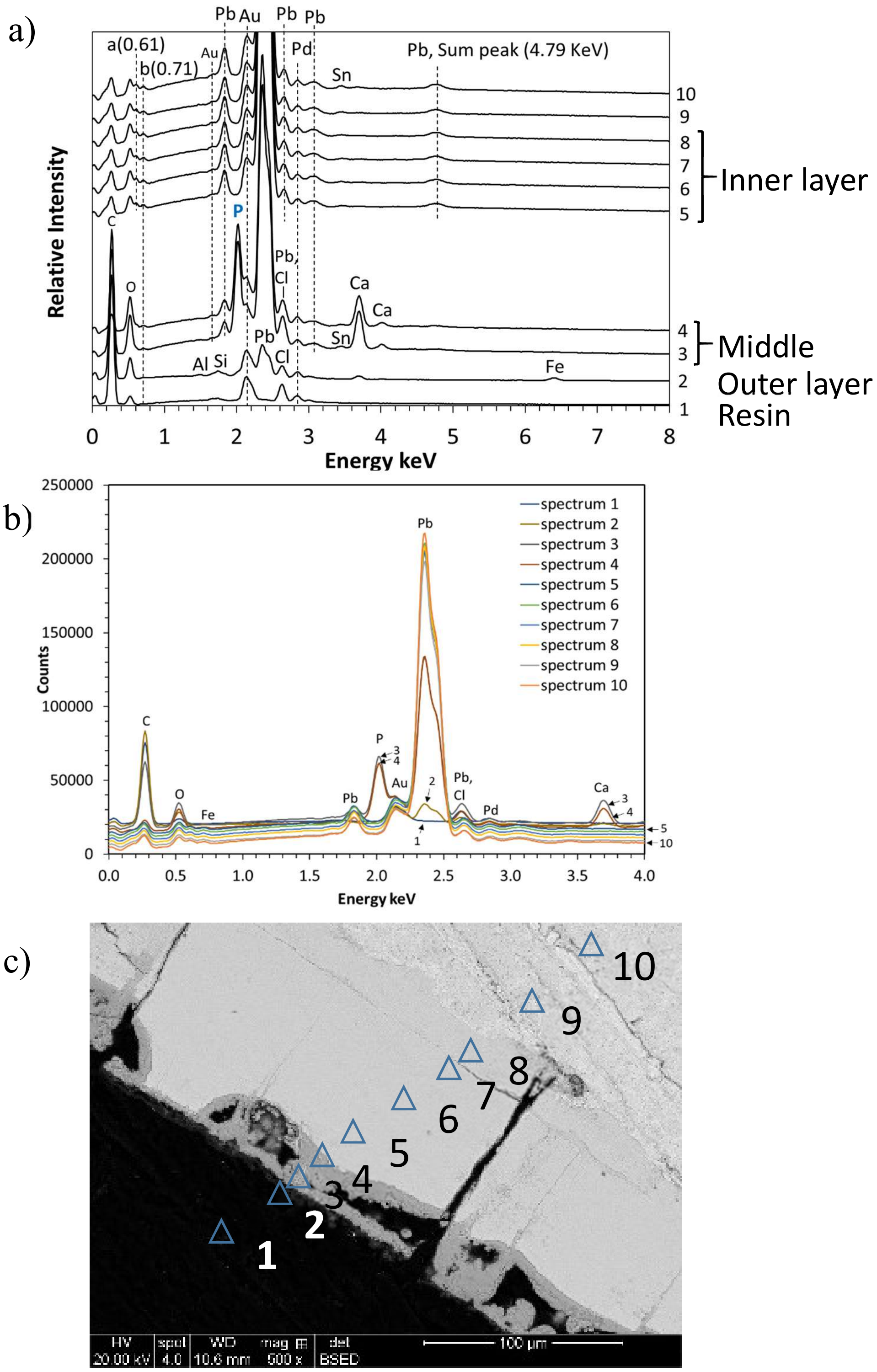

Figure S9. EDS analysis of the mineral scale showing P and Ca to be concentrated in the middle layer. a, b. EDS spectra (point analysis) and c. Back scattered SEM image of a cross-sections of polished lead pipe. The peaks at 2.62 KeV were lead or chlorine. Those in lines 1 and line 2 were probably chlorine from the epoxy resin, Those in lines 3 and line 4 were chloride and/or lead. Those in lines 5 to 10 were lead. Graphs a and b are the same data plotted in a different order, with a different spacing between the graphs. The middle layer, which contained the spherulites, is given by points 3 and 4.

Text S1. Interpretation of the EDS Maps

The purpose of the maps in Figures 2 and 4 of the main text was to show where the elements of Pb, P, Ca and Fe were concentrated. The maps from Figure 2 are presented below. The elements P and Ca are concentrated in the middle layer, Fe is concentrated in the outer layer and Pb is present throughout all layers as well as the Pb metal.

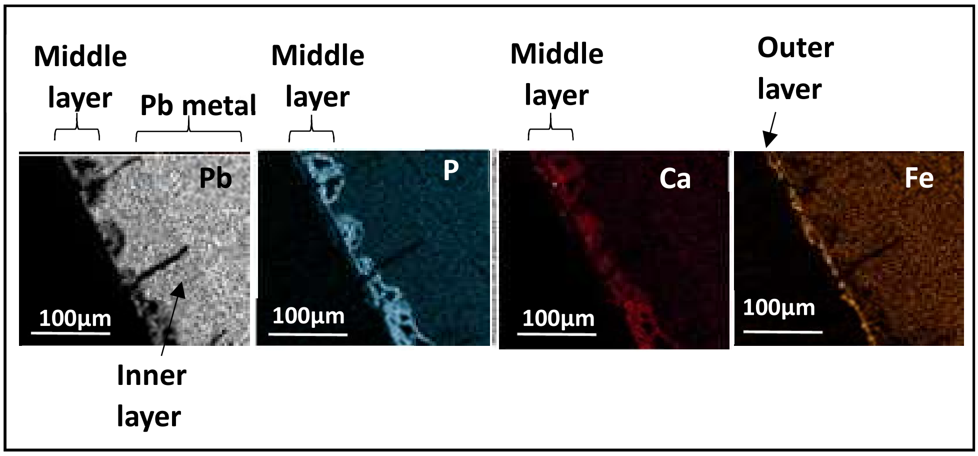

The maps also show minor amounts of P and Ca in the inner layer and Pb metal. However, there were no peaks for P and Ca in the EDS spectra of these regions (Figure S9) and only background counts were observed. Therefore, the light shades of blue and red in the maps of P and Ca represent the background radiation. A similar explanation can be given for the apparent trace amount of Fe in the middle layer and Pb metal. The EDS spectrum of the outer layer (Figure S9) shows that Fe is concentrated in the outer layer and isn't detectable in the middle, inner and Pb metal. The fact that the peak is small, even in the outer layer, suggests that it was close to the limit of detection and the small number of counts would increase the error associated with the peak. Therefore, the light shade of yellow / orange in the middle, inner and Pb metal the map of Fe represents the background radiation. Improvements to the maps could have been made by increasing the dwell time on each pixel (increasing the number of counts) and by accounting for and removing the background counts.

It is also possible that low concentrations of P, Ca and Fe in the inner layer and Pb metal was an artefact of the polishing process. In the case of P, this was present in the tap water used to polish the samples, at a concentration of ≈ 3 mg/L phosphate and this may have reacted with the freshly polished lead metal surfaces to give a false reading for the inner layer and Pb metal. Alternatively, some Fe, P and Ca containing material removed from the outer and middle layers during polishing may have been deposited on other parts of the sample. The possibility of contamination during polishing is why an argon ion mill cross section polisher was used in a recent study[5], however this piece of equipment wasn't available in this study. Lastly, in the case of P, the trace amount seen in the inner and Pb metal might be due to the base of the large Pb peak at 2.36 KeV overlapping with the P peak at 2.02 KeV.

Although they have their limitations, the maps shows visually that P and Ca are concentrated in the middle layer and Fe is concentrated in the outer layer.

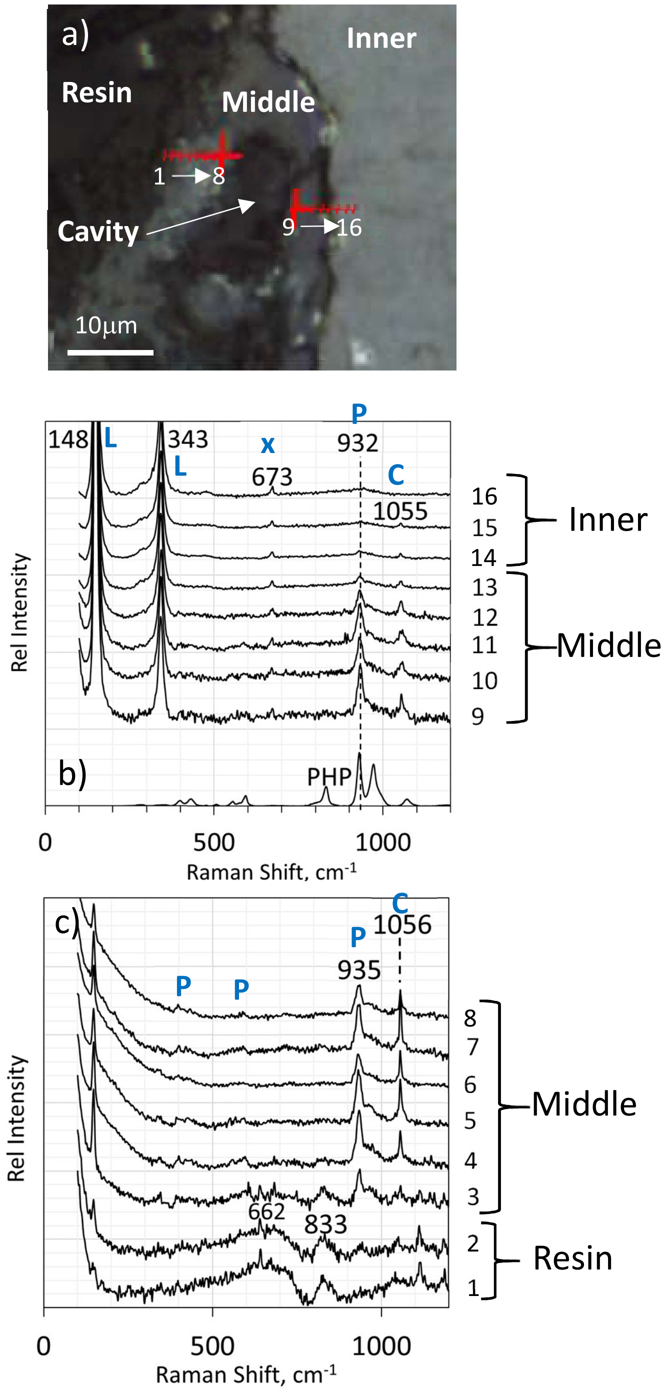

Figure S10. Raman spectroscopy of the mineral scale, analysis 1, showing that lead phosphate and lead carbonate are concentrated in the middle layer. a) Optical microscope image of a cross-section of polished lead pipe, b,c) Raman spectra across the mineral layer. Points 3 – 13 were from the middle layer. Peaks corresponding to lead oxide (litharge), lead phosphate and lead carbonate are labelled L, P and C. Power = 0.4mW, λ=532nm, 5 exposures per point, 2s per exposure. The spectra for the lead calcium apatite mineral phosphohedyphane $Ca_2Pb_3(PO_4)_3Cl$ (PHP), is also shown (RRUFF database (R060733))[4]. The identity of the peaks at 673 cm-1 in fig 'b' (marked x) was not known. Standard spectra are shown in Figure S12.

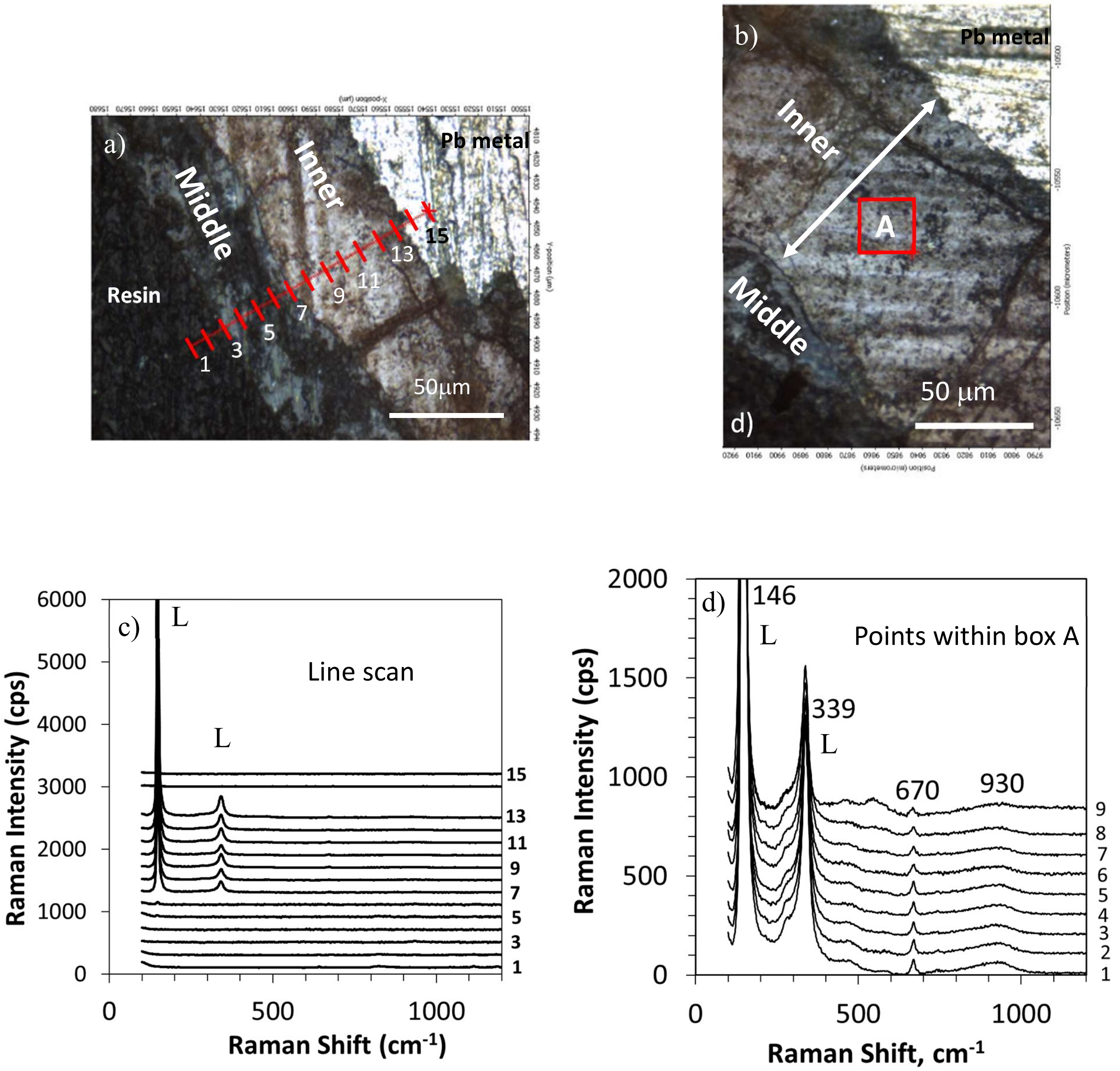

Figure S11. Raman spectroscopy of the mineral scale, analysis 2, showing that lead oxide (litharge, L) is concentrated in the inner layer. a,b) optical microscope images of cross-sections of polished lead pipe, c) Raman spectra for points 1–15 in fig 'a', d) Raman spectra for 9 different points in box A in 'fig b'. Power = 3.0mW, λ=532nm, 5 exposures per point, 2s per exposure. The identity of the peaks at 670 cm-1 in fig 'b' was not known. Standard spectra are shown in Figure S12.

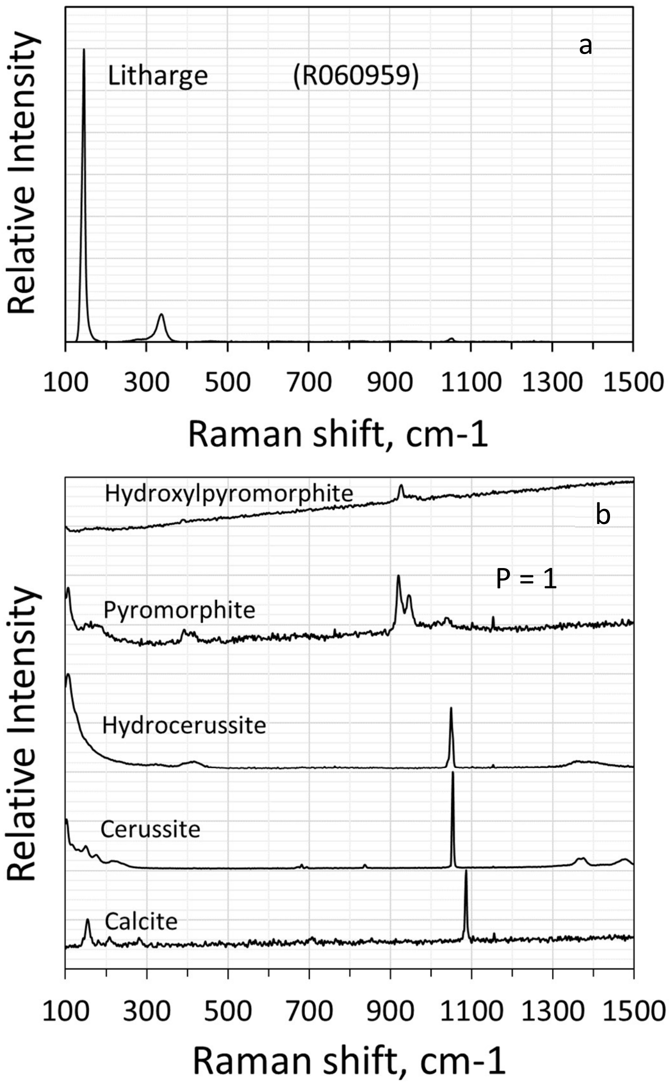

Figure S12. Raman spectra of standards and pure lead minerals. a) Standard spectra of litharge taken from the RRUFF database (R060959), b) Raman spectra of pure samples of lead phosphate, lead carbonate and calcite minerals taken at a 532nm and a power of 1.0 mW, using the same Raman microscope as that used to analyse the lead pipe mineral scale.

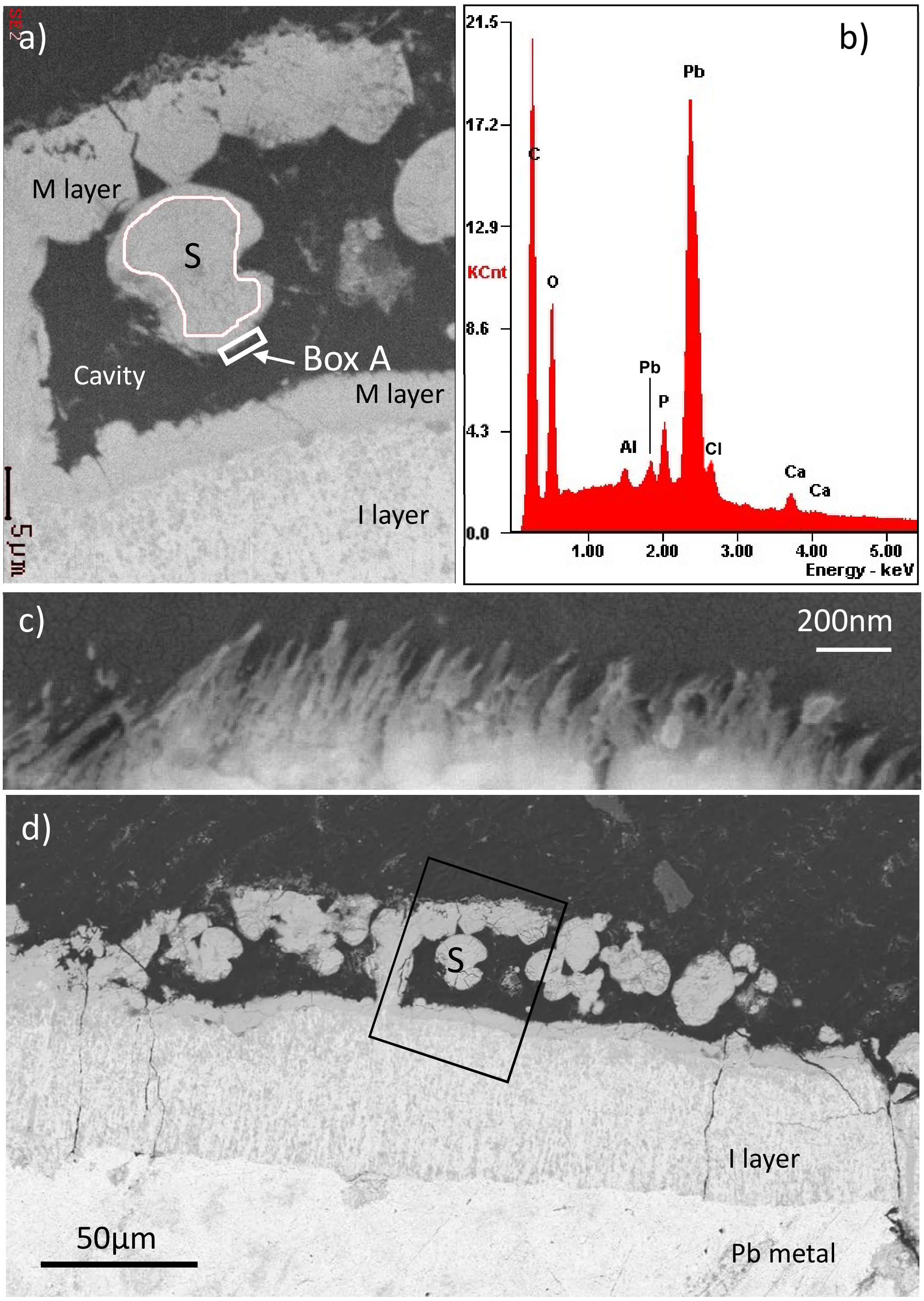

Figure S13. Additional backscattered SEM images of lead pipe spherulites. The images are of polished sections of mineral scale. The spherulite marked S is the one shown in Figure 4 of the main text. The EDS spectra of the spherulite (within the white lined area) is shown in 'Fig b'. The outer surface of the spherulite S (box A) is magnified in Fig 'c' to show the protruding needles. Fig 'd' is a low magnification image showing the surrounding scale.

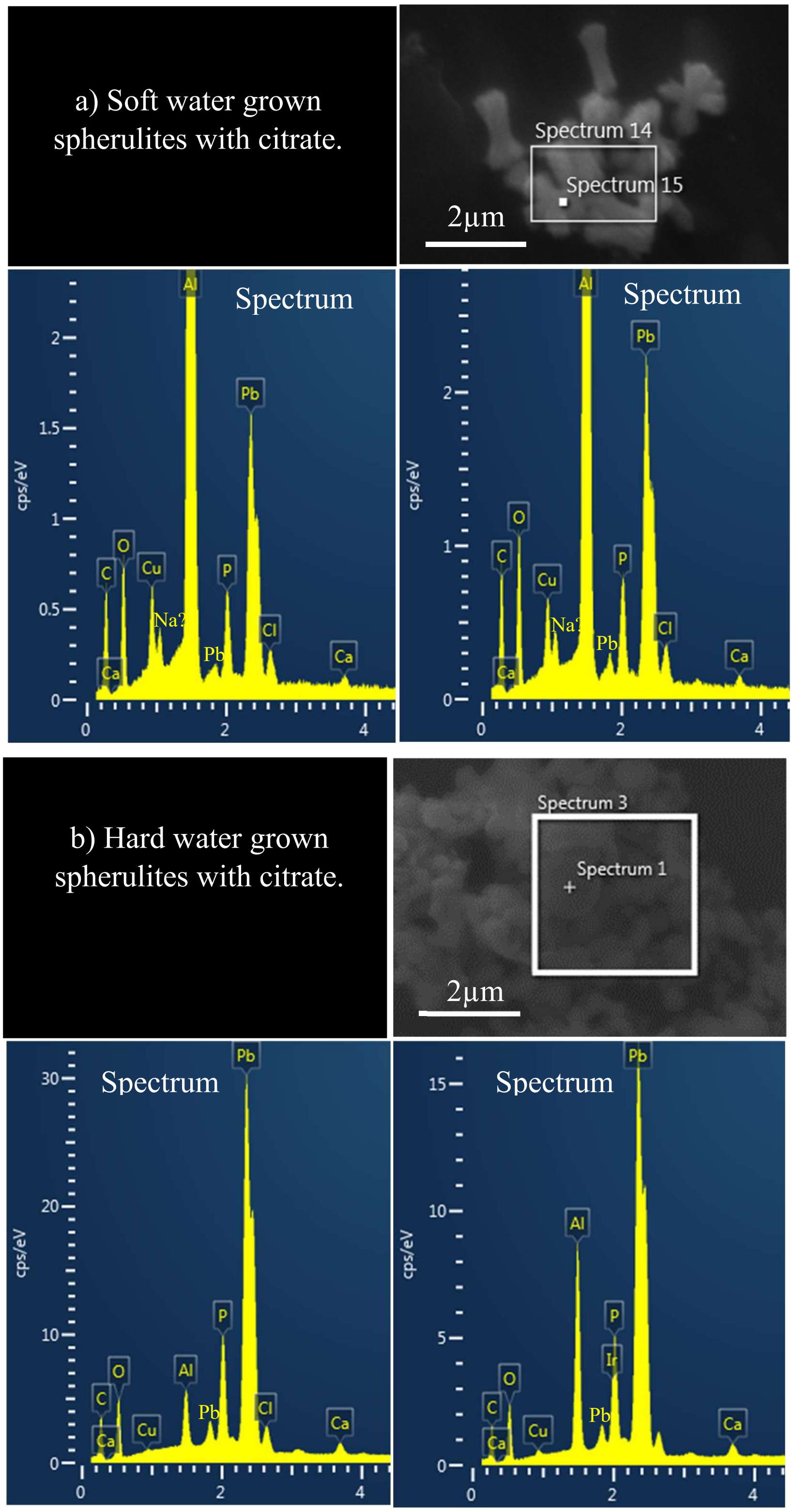

Figure S14. Elemental EDS spectra and secondary electron images of laboratory grown spherulites. The spherulites were grown in the laboratory by adding hydrocerussite to a) soft water and b) hard water both containing citrate and phosphate. The spherulites contained Pb, P, Ca and possibly Cl. Al and Cu came from the aluminium SEM stubs and the peak at 1.05 might be Na from the sodium citrate. The peaks for C and O are not reliable. The SEM images are in plan-view.

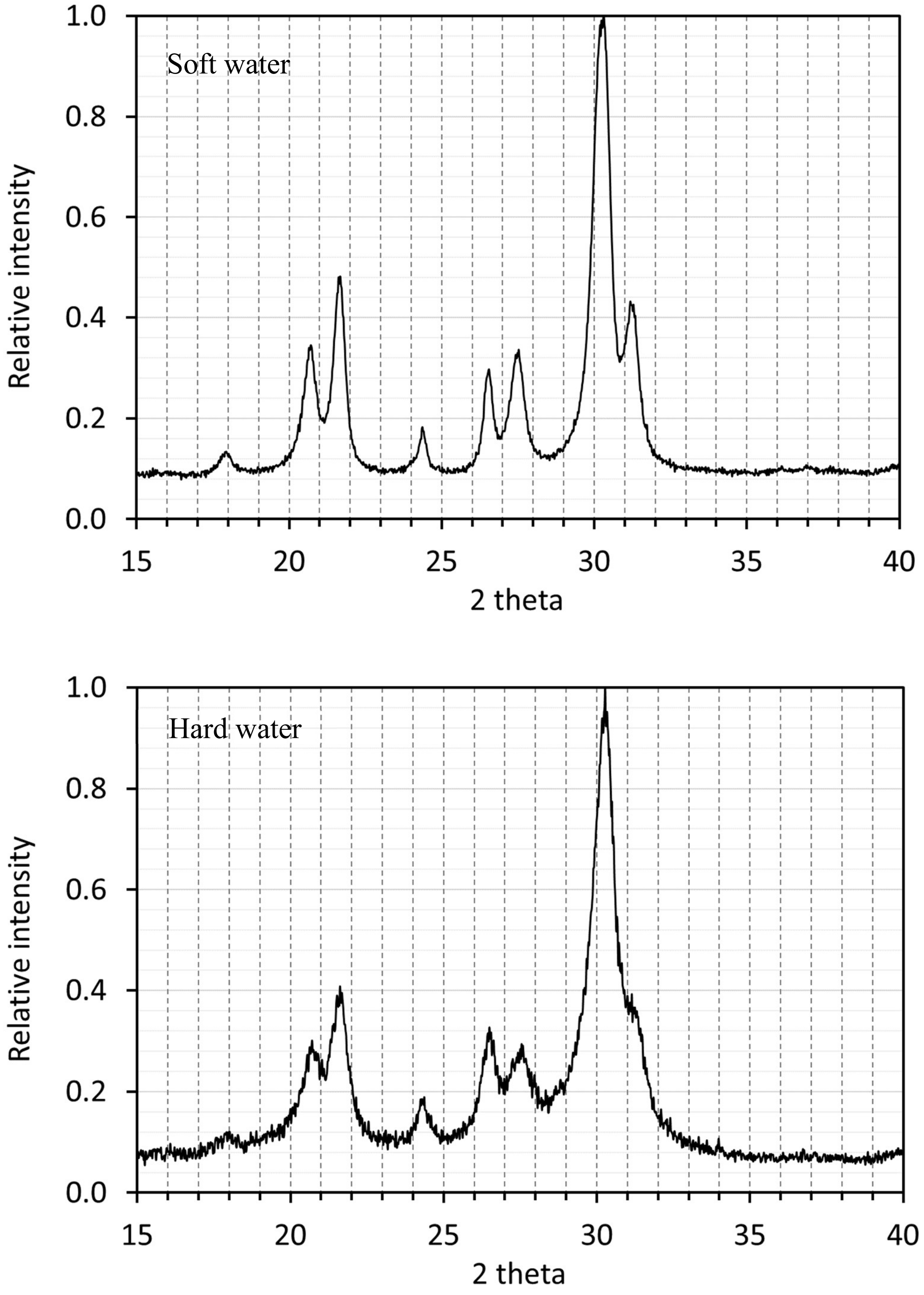

Figure S15. XRD spectra of laboratory grown spherulites. These were grown by adding hydrocerussite to soft and hard waters containing phosphate and citrate at pH 5.5. The peaks in both spectra index for lead apatite. The same set of peaks can be seen in the XRD spectra for pyromorphite, hydroxylpyromorphite and phosphohedyphane shown in Figure S4.

a)

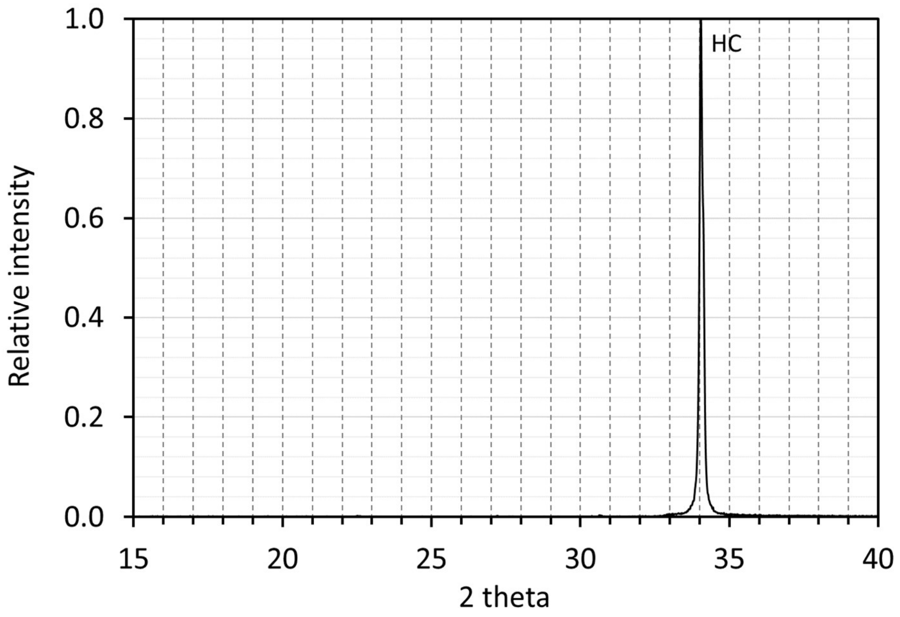

b)

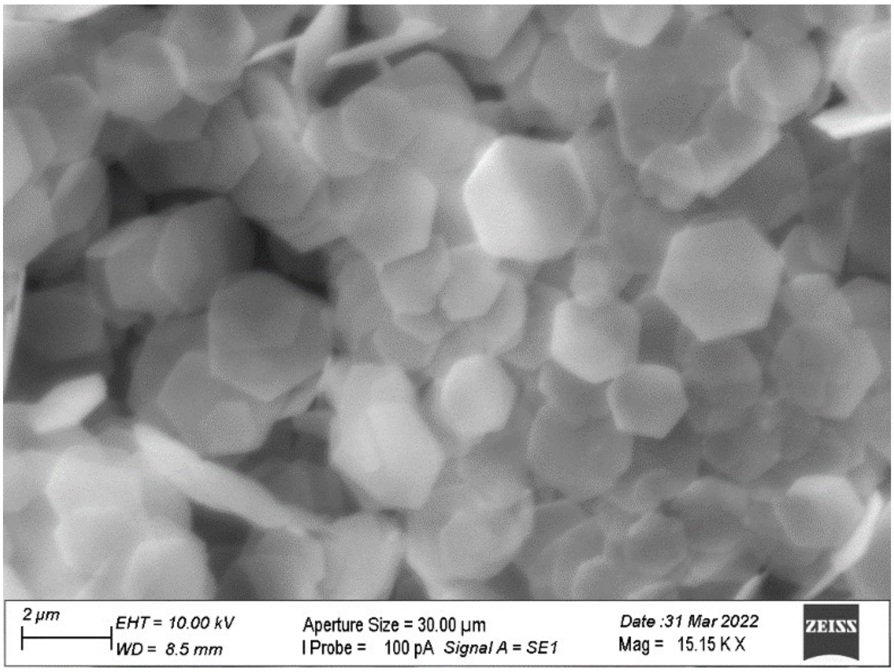

Figure S16. XRD pattern and secondary electron SEM image of hydrocerussite used to make spherulites. a) XRD pattern showing a single peak at $2\theta = 34.0°$ for the {110} planes of hydrocerussite. The single peak was due to preferential orientation in which the large hexagonal (001) faces were parallel to the surface of the silicon sample holder. b) SEM image of the hydrocerussite crystals.

a)

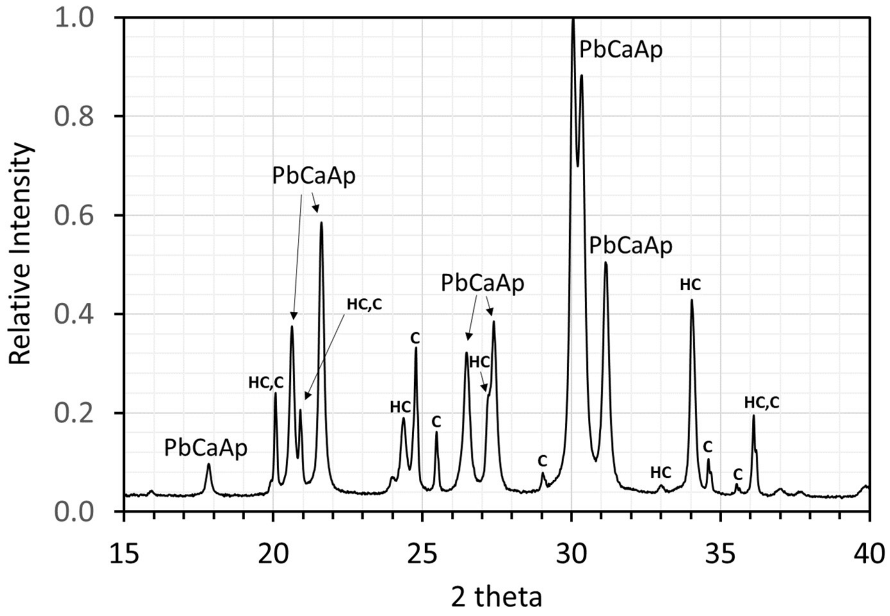

b)

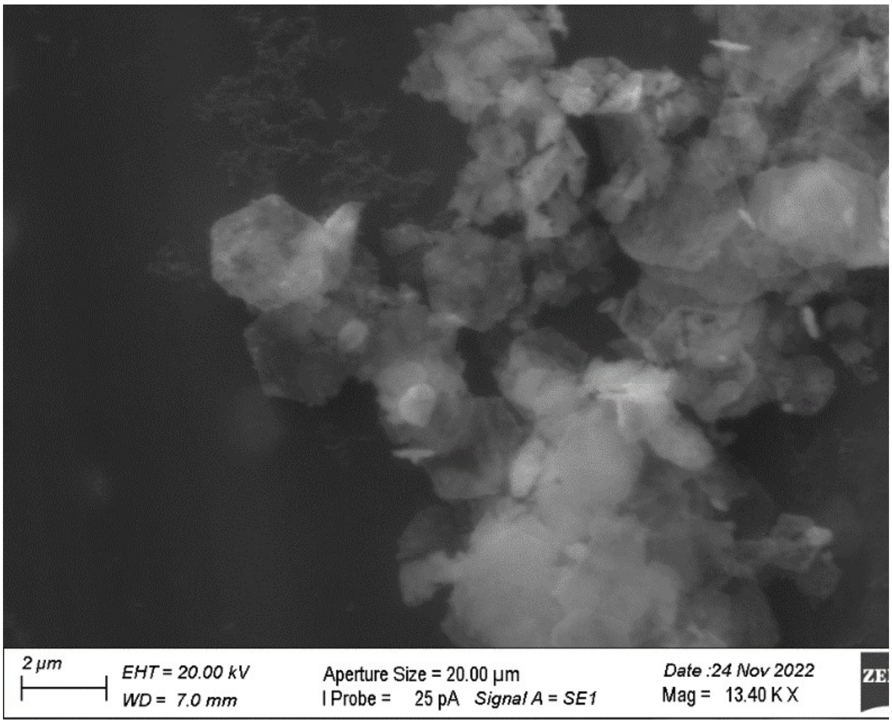

Figure S17. XRD pattern and secondary electron SEM image of crystals from the soft water control expts. a) XRD pattern of crystals in solution after adding hydrocerussite to a pH 5.5 soft water containing 10mg/L phosphate and10mg/L chloride and no citric acid. Samples were taken after 1 day. The XRD pattern indicated that there was a mixture of lead calcium apatite, hydrocerussite and cerussite, which in turn suggested that the original hydrocerussite had mostly dissolved reprecipitating as lead calcium apatite and to a lesser extent cerussite. b) SEM image of the crystals. Spherulites were not observed in this or other SEM images of the sample. Instead, there was a mixture of hexagonal shaped crystals, which were probably partially dissolved hydrocerussite and fine grain nano sized particulates, which could not be resolved.

a)

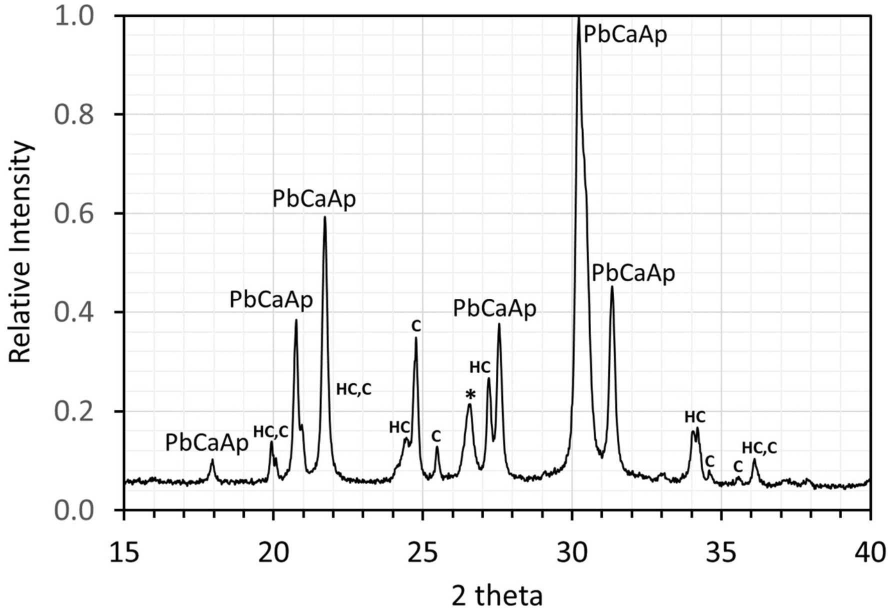

b)

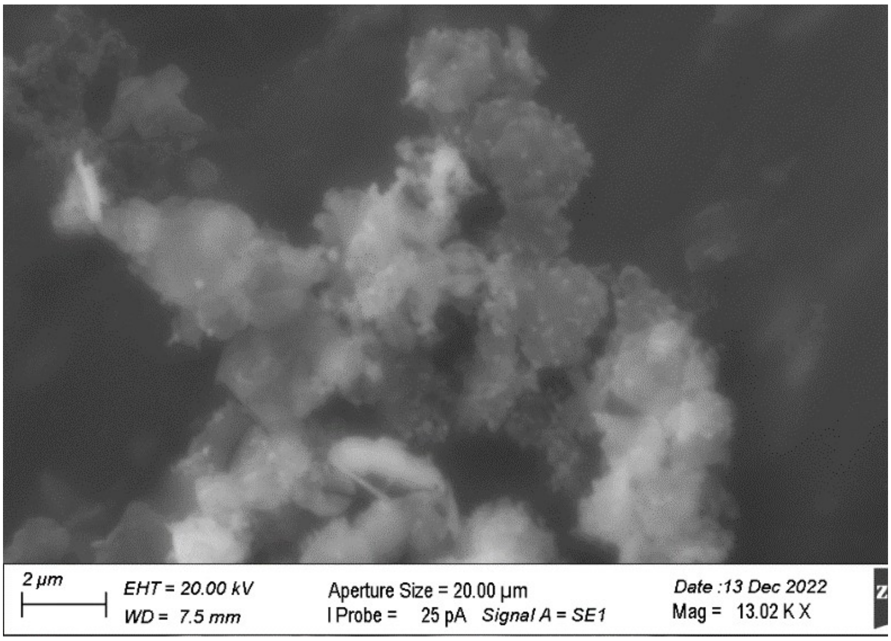

Figure S18. XRD pattern and secondary electron SEM image of crystals from the hard water control expts. a) XRD pattern of crystals in solution after adding hydrocerussite to a pH 5.5 hard water containing 10mg/L phosphate and10mg/L chloride and no citric acid. Samples were taken after 1 day. The XRD pattern indicated that the sample was mostly lead calcium apatite together with a small amount of hydrocerussite and cerussite. The peak labelled with a * is probably a peal for calcium lead apatite. But it is broader than the other peaks and occurs at the same place as a peak for plumbonacrite and so there is uncertainty about its identity. b) SEM image of the crystals. Spherulites were not observed in this or other SEM images of the sample. The crystals appeared hexagonal in outline with small particulates on their surfaces. The particulates were probably lead calcium apatite and the retention of the hexagonal shape indicates that the replacement may have been psueodomorphic.

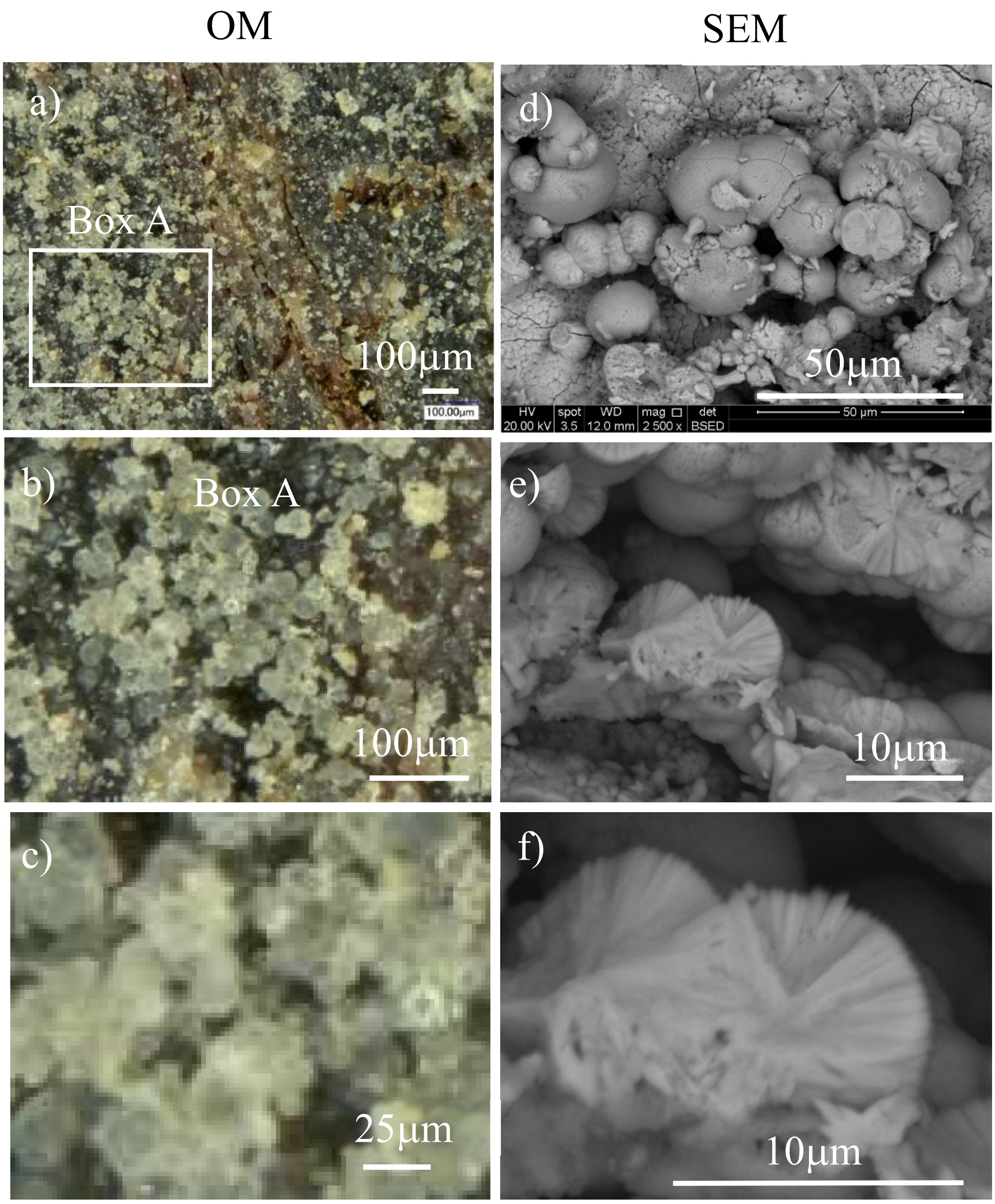

Figure S19. OM and secondary electron SEM images of spherulites observed on an additional pipe. The pipe is referred to as P7. Figures a, b and c are enlargements of each other. Figure d is another location on the pipe scale. Figure f is an enlargement of Figure e. The water supplying this pipe had an average alkalinity of 150 mg/l CaCO3, a pH of 7.6 and a phosphate concentration of 1.0 mg/l as P. The XRD pattern of the mineral scale contained peaks for lead calcium apatite and is published elsewhere[6].

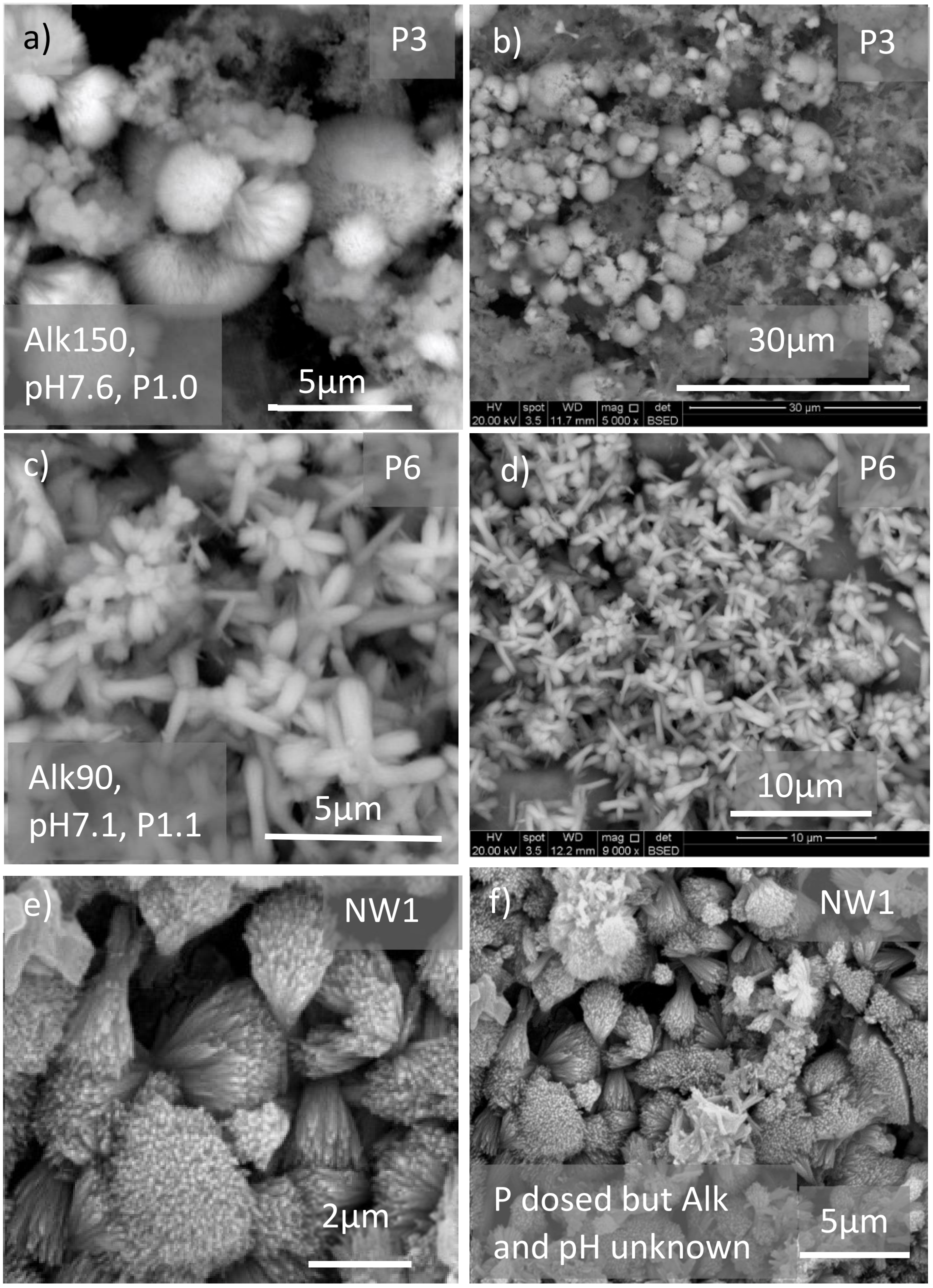

Figure S20. Secondary electron SEM images of spherulitic lead calcium apatites observed on 3 more pipes. Pipes P3, P6 came from Yorkshire Water and pipe NW1 came from Northumbrian Water. Their alkalinities, pH and phosphate concentrations are shown on each image. Figures a, c and e are enlargements of figures b, d and f. XRD patterns of P3 and P6 all contained peaks for lead calcium apatite and have been published elsewhere[6].

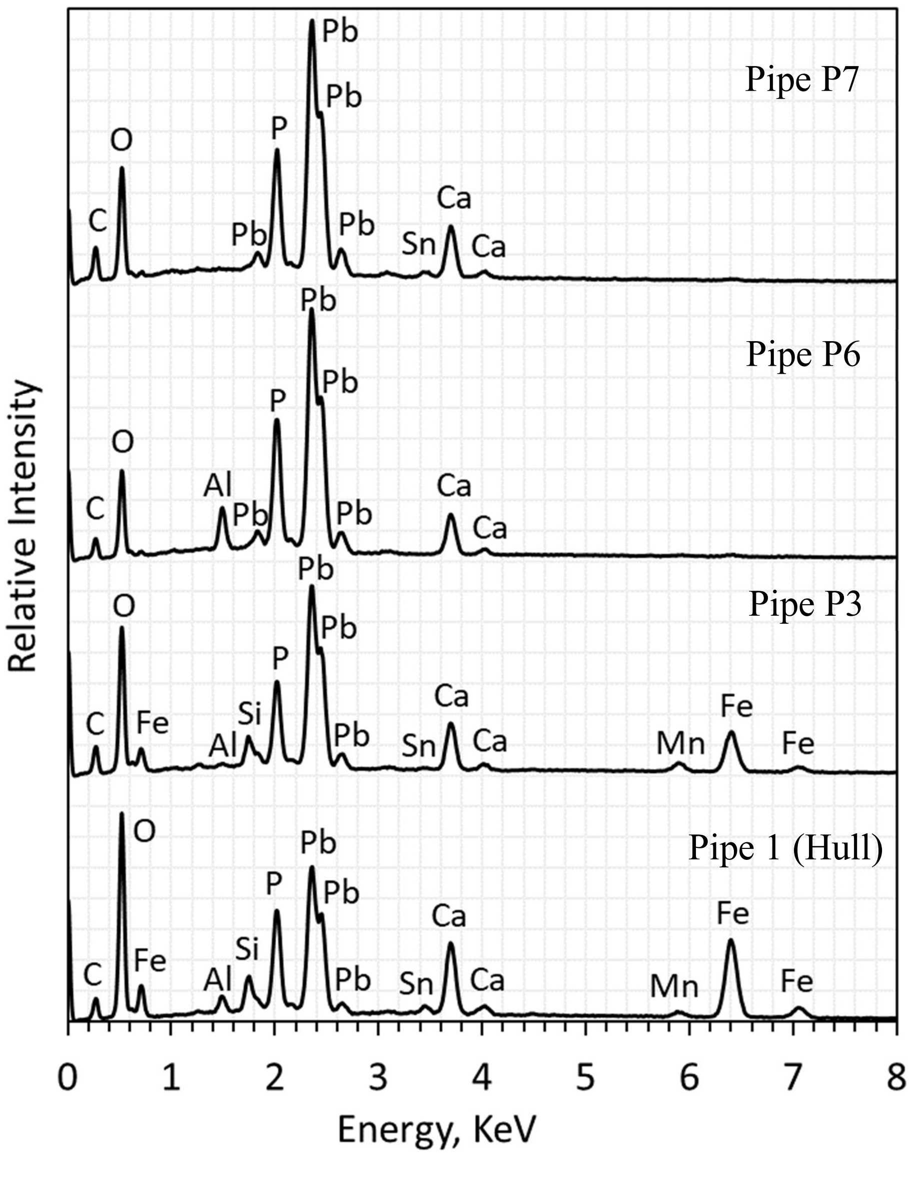

Figure S21. EDS spectra of spherulites from pipes P1, P3, P6 and P7. Pipe P1 is the pipe analysed in the main paper and pipes P3, P6 and P7 are those shown in Figures S19 and S20. The spectra were taken of samples in plan-view (not polished). The elements Pb, Ca and P are consistent with the presence of lead calcium apatite. The Al, Si and Fe peaks were from the outer layer.

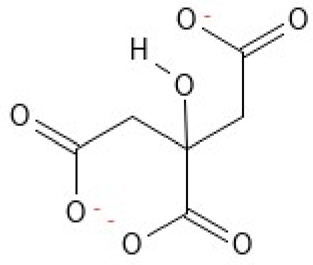

Figure S22. Structure of the citrate ion. It has three carboxylate groups, which can bind to Pb^{2+} ions and one hydroxide group.

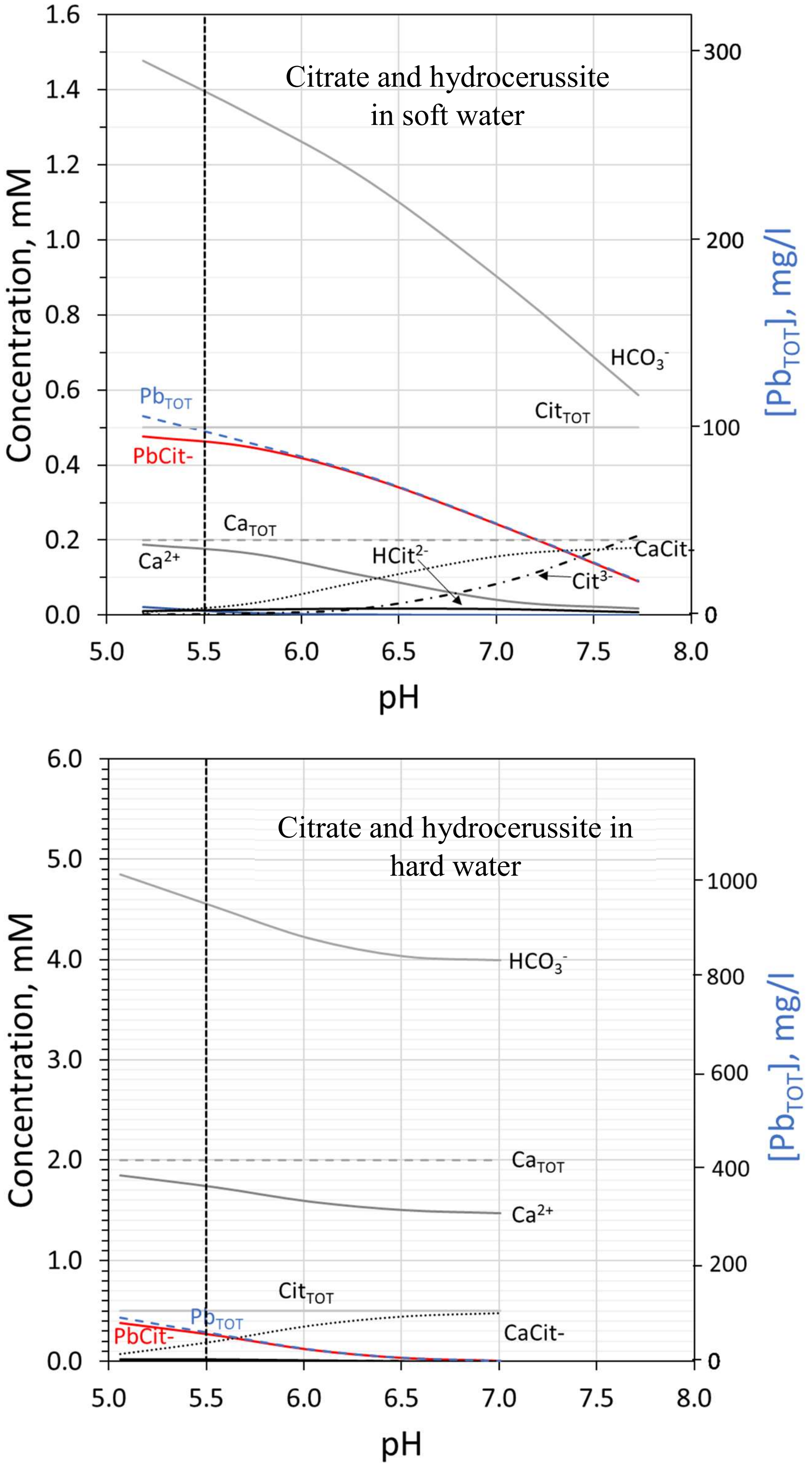

Figure S23. Speciation diagrams for 0.5mM citrate in equilibrium with in soft and hard waters containing hydrocerussite. The diagrams were calculated using the stability data in Table S2 and the PHREEQC input files in Figure S2. The graphs are a guide only as the accuracy of the stability data is not known. At pH 5.5, the concentrations of PbTOT (total) in soft water containing citrate is 100 and in hard water it is 57 mg/l. The very high concentrations are due to the stability of the PbCit- ion.

Calculation S1. Calculation showing that the spherulites are not caused by sample preparation.

This calculation shows the number of phosphohedyphane $Ca_2Pb_3(PO_4)_3Cl$ spheres that could form in a film of water remaining on a lead water pipe after draining.

V: volume of tap water left in the pipe after draining for 5 minutes, cm^3

σ : density of phosphohedyphane, gcm^{-3}

d : diameter of the PHP spheres, µm

A_P : atomic weight of phosphorus

A_{PHP} : atomic weight of phosphohedyphane

[P] : concentration of phosphorus in tap water, mg/l

L : length of pipe section, m

D : internal dimeter of pipe, m

N : Number N of PHP spheres in pipe of length L

t : thickness of film of water left in pipe after draining for 5 minutes, m

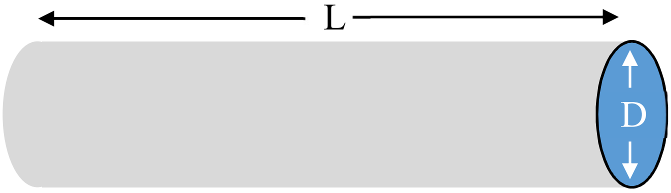

Step 1. Mass of P in tap water left in the pipe after draining for 5 minutes

Mass of P in $1cm^3$ = $[P]/1.00x10^6$ grams

Mass of P in Vcm^3 = $V.[P]/1.00x10^6$ grams

Step 2. Maximum mass of PHP that can be formed

Moles of P in the tap water left in the pipe after draining for 5 minutes :

Moles = mass / RAM

Moles of P in Vcm^3 = $(V.[P]/1.00x10^6)/30.97$

Moles of P in Vcm^3 = $3.23x10^{-8}$ V.[P]

Formula of phosphohedyphane : $Pb_3Ca_2(PO_4)_3Cl$

Maximum number of moles of phosphohedyphane that can be produced from P in the water : $3.23x10^{-8}$ V.[P] / 3

Max. moles PHP of P in Vcm^3 = $1.08x10^{-8}$ V.[P]

RMM of PHP = 1022.12

Max. mass PHP of in Vcm^3 = moles x RMM

Max. mass PHP in $Vcm^3 = 1.08x10^{-8}$ V.[P] x 1022.12

Max. mass PHP in $Vcm^3 = 1.10x10^{-5}$ V.[P]

<u>Step 3. Number N of 10μm diameter PHP spheres in pipe of length L</u>

Volume of PHP in cm^3 = mass / σ

Volume of PHP in $cm^3 = 1.10x10^{-5}$ V.[P]. σ^{-1}

Volume of PHP in $m^3 = 1.10x10^{-5}$ V.[P]. σ^{-1}. $1.00x10^{-6}$

Volume of PHP in $m^3 = 1.10x10^{-11}$ V.[P]. σ^{-1}

Volume of a PHP sphere in $m^3 = 4/3\Pi(d/(2x10^6))^3$

Volume of a PHP sphere in $m^3 = 4/3\Pi d^3.(1.25x10^{-19})$

Volume of a PHP sphere in $m^3 = 1.67x10^{-19}\Pi.d^3$

Number of PHP spheres in pipe = Volume of PHP divided by volume of a PHP sphere

Number of PHP spheres in pipe = $1.10x10^{-11}$ V.[P]. σ^{-1} / $1.67x10^{-19}\Pi.d^3$

Number of PHP spheres in pipe = $6.59x10^7$.V.[P]. σ^{-1}. $\Pi^{-1}d^{-3}$

<u>Step 4. Number of PHP spheres in a 100μm x 100μm plan-view SEM image</u>

Surface area inside the pipe in m^2 = ΠDL

Number of PHP spheres per $m^2 = 6.59x10^7$.V.[P]. σ^{-1}. $\Pi^{-1}d^{-3}.(\Pi DL)^{-1}$

Number of PHP spheres per $m^2 = 6.59x10^7$.V.[P]. σ^{-1}. $\Pi^{-2}.d^{-3}.D^{-1}.L^{-1}$

Planar surface area in an SEM image of dimensions 100μm x 100μm = $1x10^{-8}$ m^2

Number of PHP spheres in an area 100μm x 100μm

= $1x10^{-8}.6.59x10^7$.V.[P]. σ^{-1}. $\Pi^{-2}.d^{-3}.D^{-1}.L^{-1}$

Number of PHP spheres in an area 100μm x 100μm = $6.59x10^{-1}$.V.[P].$\sigma^{-1}.\Pi^{-2}.d^{-3}.D^{-1}.L^{-1}$

Number of PHP spheres in an area 100μm x 100μm = [$6.59x10^{-1}$.V.[P]] / [$\sigma.\Pi^2.d^3$.D.L]

<u>Step 5. Number of PHP spheres in a 1000μm x 1000μm plan-view SEM image</u>

Planar surface area in an SEM image of dimensions 1000μm x 1000μm = $1x10^{-6}$ m^2

Number of PHP spheres in an area 1000μm x 1000μm

= $1x10^{-6}.6.59x10^7$.V.[P]. σ^{-1}. $\Pi^{-2}.d^{-3}.D^{-1}.L^{-1}$

Number of PHP spheres in an area 100μm x 100μm = 65.9xV.[P].$\sigma^{-1}.\Pi^{-2}.d^{-3}.D^{-1}.L^{-1}$

Number of PHP spheres in an area 100μm x 100μm = [65.9 V.[P]] / [$\sigma.\Pi^2.d^3$.D.L]

<u>Step 6. Area occupied by the spheres if they were all grouped together in a square packed array.</u>

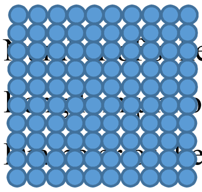

[illegible]eres on each side of the square = √N

[illegible]f each side of square = √N.d

[illegible] square area containing the spheres = √N.d

Step 7. Relationship between the volume of water remaining in the pipe and its thickness as a film

Volume of water in the film = Area of film annulus x Length

Area of annulus = = $\Pi D^2/4 - \Pi(D-2t)^2/4$

Area of annulus = $\Pi/4(D^2 - (D-2t)^2)$

Area of annulus = $\Pi/4(4Dt-4t^2)$

Area of annulus = $\Pi(Dt-t^2)$

Volume of water in the film = $\Pi(Dt-t^2)L$

$V = 1.00x10^6.\Pi(Dt-t^2)L$

Example

In this example we assume a simple situation in which a film of water of thickness of 1mm was left in the pipe after it had been cut, which corresponds to a volume of 2.7cm^3. The length of the pipe was set to 0.1m (10cm). The calculation assumes that all the spherulites were of the same diameter (10µm) and that they were 1 layer thick. The internal diameter of the pipe was set to 0.0095m (3/8 inch).

V = 2.7cm3, [P] = 1.0 mg/l, σ of PHP = 5.92gcm^{-3}, d = 10µm, D = 0.0095m, L = 0.1m

Step 1. Mass of P in tap water left in the pipe after draining for 5 minutes

Mass of P in Vcm^3 = V.[P]/1x10^6 grams

Mass of P in Vcm^3 = (2.7 x 1.0)/1x10^6 grams

Mass of P in Vcm^3 = 2.7x10^{-6}g or 1.0µg

Step 2. Maximum mass of PHP that can be formed

Max. mass PHP of P in Vcm^3 = 1.10x10^{-5} V.[P]

Max. mass PHP of P in Vcm^3 = 1.10x10^{-5}.(2.7x1.0)

Max. mass PHP of P in Vcm^3 = 3.00x10^{-5}g or 30.0 µg

Step 3. Number of 10µm diameter PHP spheres in pipe

Number of 10µm diameter PHP spheres in pipe = 6.59x10^7.V.[P]. σ^{-1}. $\Pi^{-1}d^{-3}$

Number of 10µm diameter PHP spheres in pipe = [6.59x10^7.(2.7x1.0)] / [5.92xΠx10^3]

Number of 10µm diameter PHP spheres in pipe = 1.78x10^8 / 1.86x10^4

Number of 10µm diameter PHP spheres in pipe = 9570

Step 4. Number of 10µm diameter PHP spheres in a 100µm x 100µm SEM image

Number of 10µm diameter PHP spheres in an area 100µm x 100µm = [6.59x10^{-1}.V.[P]] / [$\sigma.\Pi^2.d^3$.D.L]

Number of 10μm diameter PHP spheres in an area 100μm x 100μm = $6.59x10^{-1}$(2.7x1.0)/ 55.5

Number of 10μm diameter PHP spheres in an area 100μm x 100μm = $3.21x10^{-2}$ or 0.032

<u>Step 5. Number of 10μm diameter PHP spheres in a 1000μm x 1000μm SEM image</u>

Number of 10μm diameter PHP spheres in an area 1000μm x 1000μm = [65.9 V.[P]] / [$\sigma.\Pi^2.d^3$.D.L]

Number of 10μm diameter PHP spheres in an area 1000μm x 1000μm = 65.9(2.7x1.0) / 55.5

Number of 10μm diameter PHP spheres in an area 1000μm x 1000μm = 3.2

<u>Step 6. Area occupied by the spheres if they were all grouped together in a square packed array.</u>

Diameter of the square area containing the spheres = √N.d

Diameter of the square area containing the spheres = √9570 x 10

Diameter of the square area containing the spheres = 978 μm

Diameter of the square area containing the spheres = 1 mm

<u>Step 7. Relationship between the volume of water remaining in the pipe and its thickness as a film</u>

$V = 1.00x10^6.\Pi(Dt-t^2)L$

Thickness of the film = 1mm

t = 0.001m

$V = 1.00x10^6.\Pi(0.0095(0.001)-(0.001)^2)0.1$

$V = 1.00x10^6.\Pi(9.5x10^{-6}-1.0x10^{-6})0.1$

V = 2.7 cm3

<u>Conclusions</u>

The calculation shows that the maximum number of phosphohedyphane spheres of diameter 10μm that could have formed in a 10cm length of lead pipe was 9570. This assumes that all the dissolved phosphate in the remaining tap water reacted with lead to form phosphohedyphane and that the amount of lead available was very high. High concentrations might be generated by cracks that form after the pipe was drained. The 9570 spheres equates to approximately 0.032 spheres being observed in a SEM image of dimensions 100μm x 100μm or to 3.2 spheres being observed in a lower magnification SEM image of dimensions 1000μm x 1000μm. If all the spheres occurred together in one location, 1 layer thick, then the calculation shows that the area containing the spheres would have been 1mm across.

Image A (secondary electron plan view) is approximately 100μm x 100μm in dimension and many spherulites are visible, far more than the calculated value of 0.032. Image B (backscattered electron) is a polished cross-sectional view. At least 20 spherulites (8 of them are arrowed) are present. The number of spherulites present cannot be accounted for by the amount of phosphate present in the film of water. Therefore, they must have formed prior to the pipe being drained when it was supplying water to the property. The spherulites were therefore not an artefact of sample preparation.

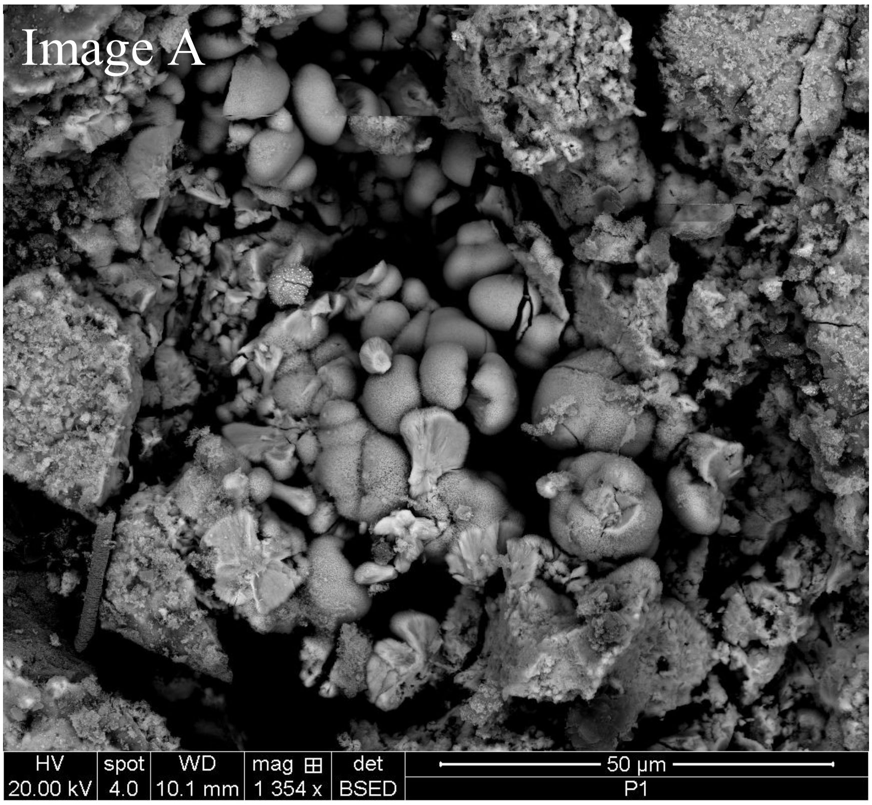

Image A
HV
20.00 kV
spot
4.0
WD
10.1 mm
mag
1 354 x
det
BSED
50 µm
P1

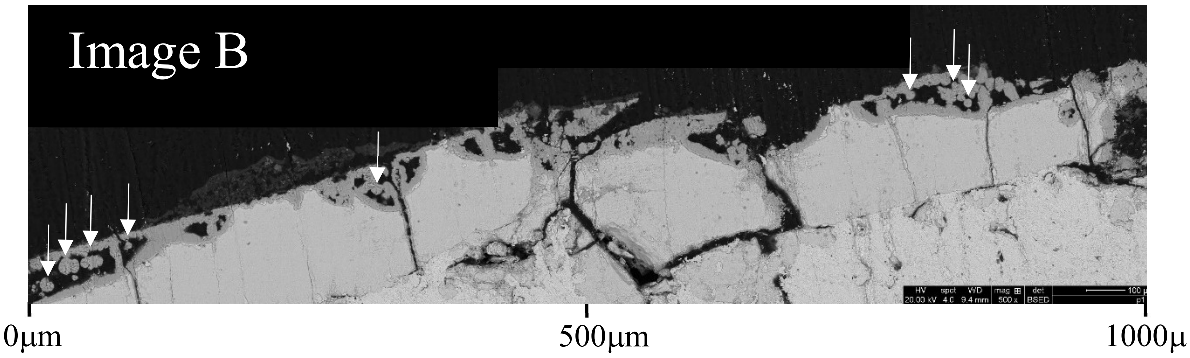

Image B
0µm
500µm
1000µ

References

1. The Water Supply (Water Quality) Regulations 2016. DWI, Ed. UK Statutory Instruments: London, 2016; Vol. No. 614 PART 8 Regulation 29, pp 1-57.
2. Geoscience, A. (1999). MINTEQA2/PRODEFA2, A Geochemical Assessment Model for Environmental Systems: User Manual Supplement for Version 4.0.
3. Smith, R.M., Martell, A.E., Motekaitis, R.J., Smith, R., & Motekaitis, R.J. (1995). NIST Critically Selected Stability Constants of Metal Complexes Database, Version 2.
4. Downs, R. T., The RRUFF Project: an integrated study of the chemistry, crystallography, Raman and infrared spectroscopy of minerals. In Program and Abstracts of the 19th General Meeting of the International Mineralogical Association, Kobe, Japan., 2006; pp 3-13.
5. Wasserstrom, L. W., Miller, S. A., Triantafyllidou, S., Desantis, M. K.; Schock, M. R. Scale Formation Under Blended Phosphate Treatment for a Utility With Lead Pipes. Journal - American Water Works Association 2017, 109 (11), E464-E478. DOI: 10.5942/jawwa.2017.109.0121.
6. Hopwood, J. D.; Derrick, G. R.; Brown, D. R.; Newman, C. D.; Haley, J.; Kershaw, R.; Collinge, M. The identification and synthesis of lead apatite minerals formed in lead water pipes. Journal of Chemistry 2016, 2016, Article. DOI: 10.1155/2016/9074062.
